# Non‐Invasive Detection, Precise Localization, and Perioperative Navigation of In Vivo Deep Lesions Using Transmission Raman Spectroscopy

**DOI:** 10.1002/advs.202301721

**Published:** 2023-06-20

**Authors:** Zongyu Wu, Binge Deng, Yutong Zhou, Haoqiang Xie, Yumin Zhang, Li Lin, Jian Ye

**Affiliations:** ^1^ State Key Laboratory of Systems Medicine for Cancer, School of biomedical engineering Shanghai Jiao Tong University Shanghai 200030 P. R. China; ^2^ Institute of Medical Robotics Shanghai Jiao Tong University Shanghai 200240 P. R. China; ^3^ Shanghai Key Laboratory of Gynecologic Oncology, Ren Ji Hospital, School of Medicine Shanghai Jiao Tong University Shanghai 200127 P. R. China

**Keywords:** depth determination, depth estimation, gap‐enhanced Raman tag, sentinel lymph node, spatially offset Raman spectroscopy, surface‐enhanced Raman scattering

## Abstract

Non‐invasive detection and precise localization of deep lesions have attracted significant attention for both fundamental and clinical studies. Optical modality techniques are promising with high sensitivity and molecular specificity, but are limited by shallow tissue penetration and the failure to accurately determine lesion depth. Here the authors report in vivo ratiometric surface‐enhanced transmission Raman spectroscopy (SETRS) for non‐invasive localization and perioperative surgery navigation of deep sentinel lymph nodes in live rats. The SETRS system uses ultrabright surface‐enhanced Raman spectroscopy (SERS) nanoparticles with a low detection limit of 10 pm and a home‐built photosafe transmission Raman spectroscopy setup. The ratiometric SETRS strategy is proposed based on the ratio of multiple Raman spectral peaks for obtaining lesion depth. Via this strategy, the depth of the phantom lesions in ex vivo rat tissues is precisely determined with a mean‐absolute‐percentage‐error of 11.8%, and the accurate localization of a 6‐mm‐deep rat popliteal lymph node is achieved. The feasibility of ratiometric SETRS allows the successful perioperative navigation of in vivo lymph node biopsy surgery in live rats under clinically safe laser irradiance. This study represents a significant step toward the clinical translation of TRS techniques, providing new insights for the design and implementation of in vivo SERS applications.

## Graphical abstract

In vivo ratiometric transmission Raman spectroscopy combined with ultrabright surface‐enhanced Raman scattering nanoparticles is demonstrated for the non‐invasive precise localization, depth estimation, and perioperative surgery navigation of deep sentinel lymph nodes in live rat models under clinically safe laser irradiance. This study represents a significant step toward the clinical translation of the transmission Raman spectroscopy technique.

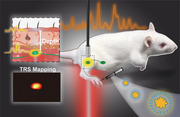

## 1 Introduction

Non‐invasive detection and localization of deep lesions via medical imaging are of significant implications for fundamental biomedical studies and clinical applications.^[^ ^1^ ^]^ In humans, lesions can be found at various depths, often several centimeters or more beneath the skin's surface. At present, surgical intervention remains the primary treatment for lesions such as malignant solid tumors and metastatic lymph nodes. Preoperative medical imaging is thus important in the current clinical procedure to locate lesions for better surgical planning. The prevailing clinical procedures for non‐invasive pre‐operative detection are X‐ray computed tomography, positron emission computed tomography (PET), and nuclear magnetic resonance imaging (MRI), but all are restricted by high costs, unwieldy instrument sizes, and limited scope for real‐time intraoperative guidance.^[^ ^2^ ^]^ In contrast, optical techniques exhibit distinct advantages, such as real‐time acquisition, non‐ionizing radiation, low cost, and ease of use.^[^ ^3^ ^]^ Therefore optical methods have been widely applied for in vivo disease diagnosis, surgery guidance, and lesion margin determination.^[^ ^4^ ^]^

The primary issue for optical approaches is shallow tissue penetration depth due to the intense absorption and scattering of photons in biological tissues.^[^ ^5^ ^]^ Typical optical methods (such as fluorescence surgical guidance systems) can only penetrate a few millimeters into the tissue.^[^ ^4^ ^]^ This makes the detection of deep lesions problematic, possibly leading to a missed diagnosis of tissue‐covered small lesions during a resection surgery. Furthermore, the complicated propagation of photons in biological tissues poses a significant challenge for the accurate determination of lesion depth. Therefore, it is significant to develop novel optical techniques for non‐invasive detection and localization of deep lesions, which could greatly assist perioperative surgery navigation, benefiting earlier disease treatment, minimally‐invasive surgery, and improved patient outcomes.^[^ ^6^ ^]^

Surface‐enhanced transmission Raman spectroscopy (SETRS) is an emerging technique to detect and monitor disease at depth in biological tissues.^[^ ^7^ ^]^ It combines functionalized surface‐enhanced Raman spectroscopy (SERS) nanoparticles (NPs) with transmission Raman spectroscopy (TRS). SERS NPs are promising imaging contrast agents with high specificity and sensitivity.^[^ ^8^, ^9^ ^]^ With its molecular fingerprint spectrum, SERS can overcome the autofluorescence background of biological tissues, allowing for multiplexed detection.^[^ ^10^ ^]^ Meanwhile, TRS is a powerful technique for detecting signals from deep layers, where Raman spectra are collected at the opposite side of the sample to illumination in a transmission configuration.^[^ ^11^ ^]^ Raman spectra are collected from the entire volume, increasing the proportion of deep photons relative to background from the shallow surface layer.^[^ ^12^ ^]^ The detection capability of TRS has been explored for the diagnosis of phantom lesions in biological tissues of centimeter‐scale thickness, much thicker than that of traditional backscattering Raman setups (only millimeter‐scale).^[^ ^12^, ^13^ ^]^ We recently also reported a home‐built TRS system to detect SERS nanotags in up to 14‐cm‐thick porcine tissues.^[^ ^14^ ^]^ As for the advances for in vivo demonstrations, it was reported that TRS can non‐invasively detect tumor lesions tagged by SERS NPs on a euthanatized mice model through thick tissues.^[^ ^15^ ^]^ We have also reported in vivo non‐invasive TRS imaging of SERS phantom lesions in an unshaved living mouse.^[^ ^14^ ^]^

Despite these recent advances, SETRS has not yet been demonstrated in the detection and surgery guidance of deep lesions in live animals. The lack of in vivo studies leads to a gap between the ex vivo demonstration and the real clinical applications for the SETRS technique. Compared to ex vivo situations, the in vivo applications are much more complex due to the highly heterogeneous environments in the living body. The first key challenge for in vivo SETRS lies in the lack of accurate localization of the lesion, which, particularly, involves the precise determination of lesion depth. This is complex for TRS detection processes, since the obtained signals are a mixture of all tissue signals in the photon propagation path. The target signal needs to be extracted first and then analyze depth of its source. Several approaches have been reported to deal with this issue, including the quantification of relative light attenuation due to the material absorption, that is, the tissue water absorption.^[^ ^13^, ^16^ ^]^ Also, the depth of a single buried layer can be determined by using multiple TRS setups,^[^ ^17^ ^]^ or by directly reading out the peak heights once knowing the original intensity of the buried nanotags.^[^ ^18^ ^]^ However, these studies were mostly conducted on ex vivo homogeneous tissues. Their reliance on pre‐existing knowledge remains a significant difficulty for accurate depth prediction of lesions in real, complex in vivo scenarios with thick, heterogeneous tissues. The second challenge for in vivo SETRS in practical clinical procedures is to realize non‐invasive detection under safe laser irradiance. High laser power densities are usually applied in TRS studies or in vivo SERS studies for better light penetration, which restricts the wider application in real clinical settings.^[^ ^19^ ^]^ Non‐invasive detection with laser irradiance below the clinical safe laser threshold, that is, below the maximum permissible exposure (MPE), is essential for real applications according to the standards for safe laser use in the clinic.^[^ ^20^ ^]^

We have recently proposed a strategy of “ratiometric Raman spectroscopy” on ex vivo tissues for depth estimation of buried SERS phantom lesions in heterogeneous tissues.^[^ ^21^ ^]^ We found a linear relationship between the natural logarithm of the intensity ratio of two Raman peaks (ln(peak‐ratio)) and the lesion depth. This is because Raman photons at different wavelengths experience varying attenuation rates due to the difference in the tissue attenuation coefficients at each wavelength. This makes the depth prediction possible by using the peak ratio values. An added benefit is that the depth prediction in both homogeneous and heterogeneous tissues can be conducted with multiple peak pairs, to improve the prediction accuracy. Overall, it remains a question whether SETRS techniques can be used in a real in vivo setting for depth estimation with the safe laser below MPE, and the studies in live animal models are highly anticipated.

**Figure 1:**
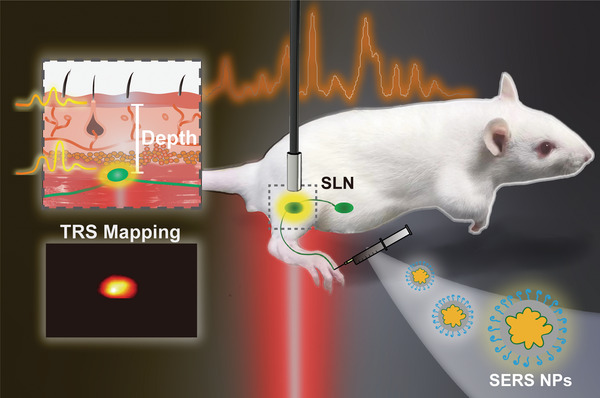
Schematic illustration of the non‐invasive detection and depth estimation of in vivo deep sentinel lymph node (SLN) on a rat model using TRS. The SERS NPs are injected on the foot pad and migrate to the SLN, which can then be non‐invasively identified from the unique Raman signal of SERS NPs using the TRS setup. Based on the Raman signal peak ratio, the depth of in vivo SLN can be determined. The location of SLN can be obtained by TRS mapping. This pre‐operative location of SLN enables the accurate removal of SLNs with minimal invasion.

In this work, we report the use of in vivo ratiometric SETRS for the non‐invasive detection, precise depth estimation, and perioperative surgery navigation of deep lesions on live rat models, using clinically safe laser irradiance (**Figure** **1**). We choose the rat's popliteal sentinel lymph node (SLN) as the deep lesion for investigation. We first construct our photosafe SETRS system, combining ultrabright SERS NP contrast agents and a home‐built TRS setup with broad beam illumination. The capability of SETRS to detect deep‐buried phantom lesions is first demonstrated on ex vivo porcine tissues, where SERS agarose gels are placed in a small tube and served as the phantom lesions. We then propose the ratiometric Raman spectroscopy method for precise lesion depth determination, based on the ratio of Raman spectral peaks. Ex vivo assessments on rat tissues show that this method does not require prior knowledge and can successfully estimate the depth of phantom lesions in either homogeneous or heterogeneous tissues with high accuracy (with the mean‐absolute‐percentage‐error of 11.8%). Based on this, we achieve non‐invasive in vivo detection and accurate localization of the 6‐mm‐deep SLNs on rat models, illustrating the potential of TRS for surgical guidance during lymph node biopsy. We further apply TRS for perioperative guidance on rat popliteal SLN under clinically safe laser irradiation (0.21 W cm^−2^), from preoperative planning, intraoperative guidance, to postoperative examination. To the best of our knowledge, this study is the first to report the application of TRS for lesion identification in a live animal model, bridging the gap between the ex vivo demonstration and in vivo practical use of TRS techniques. Our work represents a significant step toward non‐invasive localization of deep lesions in vivo and peri‐operative surgical guidance in a clinical setting using TRS techniques, providing new insights for the implementation of in vivo SERS intraoperative applications.

## 2 Result and Discussion

### 2.1 Synthesis and Characterization of SERS Nanoparticles

**Figure 2:**
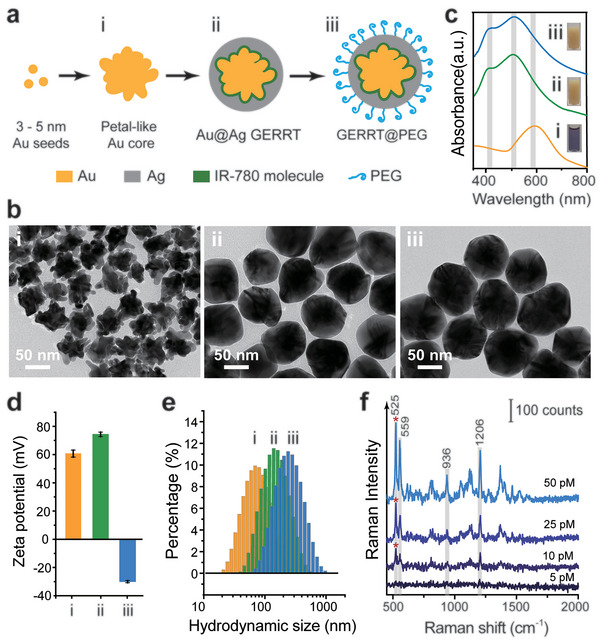
Preparation and characterization of SERS NPs. a) Scheme of SERS NP preparation. b) TEM images, c) UV–vis spectra, d) zeta potential, and e) hydrodynamic size of i) Au core NPs, ii) Au@Ag GERRTs, and iii) GERRTs with PEG layer modification. f) Raman spectra of PEG‐modified GERRTs with different concentrations. All Raman measurements were performed at the wavelength of 785 nm with 10 mW laser power and 10 ms integration time. Data were repeatedly measured three times and mean ± SD waspresented.

We first synthesized ultra‐bright SERS NPs which we refer to as “gap‐enhanced resonant Raman tags” or “GERRTs.” These are core–shell structured particles, prepared with a seed‐mediated method used in our previous studies.^[^ ^14^, ^22^ ^]^ In contrast to previous work, we developed a new protocol to make smaller metallic cores. As shown in **Figure** **2**a, the Au cores with petal‐like rough surfaces grow directly from seeds of 3–5 nm. Then, the Au cores are coated with resonant Raman reporter molecules (IR‐780) and silver (Ag) layers, to form the bimetallic Au@Ag GERRTs. Finally, thiolated polyethylene glycol (HS‐PEG) and a bifunctional PEGylation reagent with thiol and carboxylic acid (HS‐PEG‐COOH) groups are utilized to modify the surface of GERRTs. The jagged surface of the Au core is rich in plasmonic electromagnetic hot spots and provides more surface area for the Raman reporters to anchor. IR‐780 is a commercial fluorophore dye, used as a resonant Raman reporter with an absorption peak near our excitation wavelength (785 nm). The Ag coating generates further SERS enhancement and protects the embedded Raman reporters.^[^ ^23^ ^]^ The PEG modification aims to increase the stability and biocompatibility of the GERRT colloids.^[^ ^19^, ^24^ ^]^ This protocol simplifies the synthesis of the metallic core into one step, which is beneficial for mass production. Also, the current protocol allows us to keep the size of the final GERRTs under 100 nm. It was reported that NPs of over 100 nm have shown limited permeability in tumors with hypovascular and hypopermeable characteristics; the sub‐100 nm NPs were thus preferred for better tumor penetration.^[^ ^25^ ^]^ Also, a better drainage efficiency to lymph nodes could be achieved when the NP size is below100 nm.^[^ ^26^ ^]^ Also, an ideal time window (60 min) for surgical SLN biopsy was reported by using NPs with a diameter of ≈100 nm, corresponding with the interval of the NPs to migrate from popliteal lymph nodes to the sciatic lymph node.^[^ ^27^ ^]^ Therefore, it is suitable to develop NPs within and close to 100 nm for in vivo applications.

In the transmission electron microscopy (TEM) images, the petal‐like Au cores are 50 ± 5 nm in diameter (Figure 2b,i). After Ag coating, the surfaces of Au@Ag GERRTs become smooth, and their sizes increase up to 90 ± 3 nm (Figure 2b,ii). Addition of the PEG layer does not affect the morphology and size of the NPs. We would like to note that the PEG layer is too thin and transparent to be seen in the TEM images (Figure 2b,iii). In the UV–vis spectrum (Figure 2c), the localized surface plasmon resonance peak of Au core colloids is presented at ≈591 nm. For the Au@Ag GERRT colloids, a blue–shift of this peak to 511 nm is observed, and a new resonance peak seen in Ag colloids emerges at 412 nm, which proves the successful coating of the Ag shell. After the addition of PEG, the above two peaks both take a red–shift of 2–3 nm due to the refractive index of the coating. Both GERRTs and PEG‐modified GERRTs exhibit a dark yellow color in suspension (insets in Figure 2c). The surface potentials of uncoated Au cores and Au@Ag GERRTs are positive, since they are stabilized by a cationic surfactant, cetyltrimethylammonium chloride (CTAC). After PEG modification, a negative zeta potential of around −30 mV was measured, indicating the successful addition of a PEG layer and displacement of the CTAC molecules. Increases in hydrodynamic size are observed at each step of NP fabrication (Figure 2e). Finally, the PEG‐modified GERRTs were dispersed in 1% PVP aqueous solution for use, with a final hydrodynamic diameter of ≈230 nm. We continued to measure the Raman intensities of the PEG‐modified GERRTs using 785 nm excitation with laser irradiance below MPE (Figure 2f). Characteristic Raman peaks of IR‐780 molecules at 523, 939, 1203, 1367, 1528, and 1587 cm^−1^ are presented, which can be attributed to specific Raman modes. The 523 cm^−1^ peak corresponds to the out‐of‐plane substituent vibration of the phenyl ring; the 1203 cm^−1^ mode is due to the C—H in‐plane deformation;1367 cm^−1^ mode is the out‐of‐plane bending vibrations; 1528 and 1587 cm^−1^ are possibly due to the C=C in‐plane vibration and the phenyl ring vibration, respectively.^[^ ^28^ ^]^ The GERRT NPs are demonstrated to have good sensitivity with a limit of detection of down to 10 pM, using a low irradiance of 1.31 J cm^−2^ (see Supporting Information).

### 2.2 Ex Vivo Detection of a SERS Phantom Lesion Using the TRS Setup

**Figure 3:**
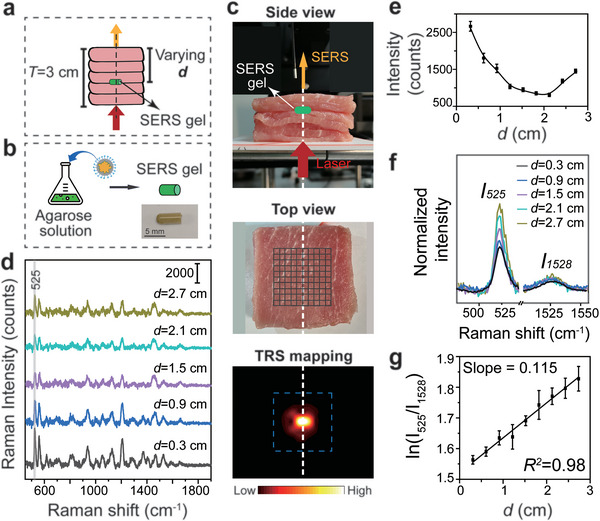
Ex vivo TRS detection of SERS phantom lesions in porcine tissues. a) Schematic illustration of a TRS mapping test on porcine muscle with an embedded SERS gel tube. The total thickness of porcine muscle was 3 cm, with each slice 3 mm thick. The SERS gel was buried in the tissue stack. The distance between the SERS gel and tissue surface was defined as the depth (***d***). b) Schematic illustration of SERS gel tube preparation. c) Experimental setup of SETRS: (top) side view, (middle) top view and the mapping area, and (bottom) TRS mapping result with the SERS gel buried in the tissue stack at a ***d*** of 0.6 cm. d) Raman spectra collected using the TRS setup at different ***d***. e) The U‐shaped relationship between Raman intensity (at 525 cm^−1^) and ***d*** (*n* = 3). f) Comparison of peak intensity between 525 and 1528 cm^−1^, and g) the corresponding natural logarithm of the peak‐pair intensity ratio at different ***d***. The laser power density was 0.21 W cm^−2^ and the integration time was 5 s. The measurements were repeated three times and mean ± SD was presented.

With ultra‐bright SERS NPs synthesized as described, we set out to construct a SETRS system combining the NPs with a home‐built TRS setup to demonstrate deep detection of phantom lesions in ex vivo biological tissues (**Figure** **3**a). As shown in Figure S1, Supporting Information, the TRS setup uses a broad‐beam laser with a wavelength of 785 nm and a low power density of 0.21 W cm^−2^. The laser is incident on the bottom surface of the target object, and Raman photons are collected by a Raman fiber optic probe connected to a Raman spectrometer. The SERS NPs are mixed into agarose gels which are filled into a small tube (Figure 3b). The SERS gel tube has a length of 5 mm and an inner diameter of 1.5 mm (Figure S2, Supporting Information). In this way, we can attain the SERS‐active material in an insulated solid state. This SERS gel tube serves as a “phantom” lesion. Then, the SERS gel tube is placed inside the ex vivo porcine muscle stack with a total thickness (*T*) of 3 cm. We first put the tube under 6‐mm of tissue (Figure 3c, top panel) and performed wide‐area TRS mapping at the tissue surface with a step size of 2 mm (Figure 3c, middle panel). The mapping result roughly indicates the shape and location of the tube, and the center of the image matches well with the horizontal position of the SERS gel (Figure 3c, bottom panel). This allows us to find the point at the center of the vertical projection of the SERS gel location on the tissue surface, that is, the center point of the image. The depth (***d***) of the SERS gel can be defined as the distance between the gel and this projected point on the tissue surface.

We further buried the SERS gel at different depths in this tissue stack. The laser, SERS gel tube, and collection probe were kept in vertical alignment. As the SERS gel was placed at each position, a distinguishable Raman signal could be detected (Figure 3d). We were able to detect signals from the lesion buried in tissues at depths of at least 2.7 cm, far exceeding that of conventional backscattering Raman spectroscopy, which is limited to only a few millimeters.^[^ ^22^ ^]^ The characteristic peak of IR‐780 at 525 cm^−1^ was selected to analyze the Raman intensity at different depths. An asymmetric U‐shaped relationship between Raman intensity and ***d*** was obtained (Figure 3e). Higher intensity is measured when ***d*** is close to 0 or 3 cm, the boundaries of the tissue stack, and lower intensity is obtained when the SERS gel is buried in the middle of the tissue stack. This is consistent with our previous reports.^[^ ^14^ ^]^

After detecting and locating the deep phantom lesions in two dimensions, we next aimed to determine their depth. Our recently proposed “ratiometric Raman spectroscopy” strategy was demonstrated to be able to estimate phantom lesion depth in ex vivo tissues.^[^ ^21^ ^]^ The two key observations underlying the strategy are: 1) Raman intensity decays exponentially in tissues, and 2) photons with different wavelengths have different effective attenuation coefficients in tissue and thus different exponential decay rates. Therefore, there is a linear relationship between the natural logarithm of the intensity ratio of two Raman peaks and the light propagation distance, that is, lesion depth. The relationship can be described by a simplified equation:

(1) $$\ln\left(\frac{I_{\lambda 1}}{I_{\lambda 2}}\right)=\Delta \mu \;\cdot d+\;m$$

Where *I*_*λ*1_ and *I*_*λ*2_ are the two selected Raman peaks with their wavelengths of *λ*_1_ and *λ*_2_. The slope of the linear model corresponds to the difference in the two attenuation coefficients in the tissue, Δ ***µ***, for the two wavelengths. *m* can be expressed by the known tissue optical parameters (see Supporting Information for more details), and is also the natural logarithm of the SERS NP peak ratios when ***d *** = 0. This linear model allows the lesion depth to be estimated from the peak intensity ratios of a Raman peak pair in the TRS spectrum. Furthermore, we can use multiple peak pairs to improve prediction accuracy. Since each peak pair gives a depth estimate, using multiple peaks gives us an overdetermined system with more equations than unknowns. Using a least square fitting method we can then calculate an improved depth prediction using all the information from the multiple peak pairs. This improves the prediction accuracy for both homogeneous and heterogeneous tissues (see Figures S4 and S5, Supporting Information).

Here, we experimentally confirmed the linear relationship using the peak intensities at 525 and 1528 cm^−1^. As shown in Figure 3f, their intensity ratio increases with depth. The expected linear relationship between the natural logarithm of the peak ratio and the depth can be seen (Figure 3g). The slope of the line was calculated to be 0.115, consistent with our previously reported Δ ***µ*** value (0.119) on porcine tissues.^[^ ^21^ ^]^ We thus achieve much higher accuracy (a millimeter scale) than in our recent work (a centimeter scale).^[^ ^21^ ^]^ Therefore, we could anticipate the application of this ratiometric Raman spectroscopy method for depth prediction of real lesions in the complicated in vivo environments of live animals.

### 2.3 Depth Estimation of Phantom Lesions in Ex Vivo Rat Tissues

**Figure 4:**
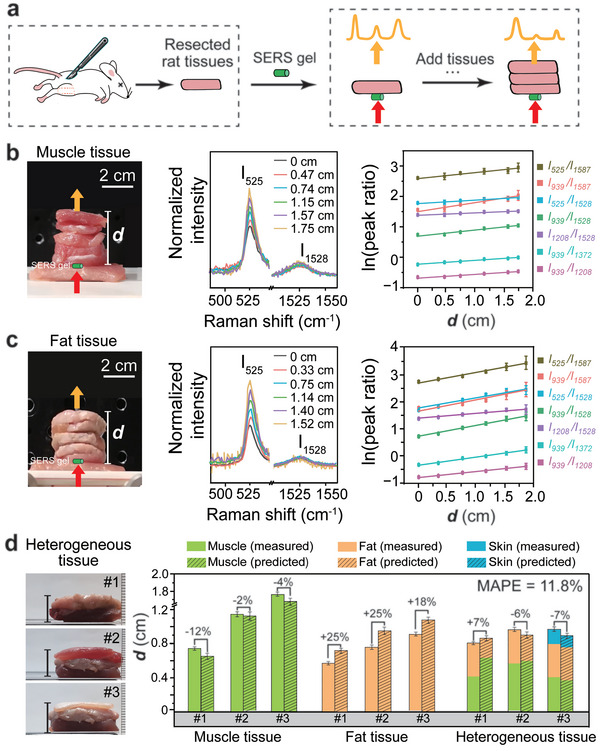
Ex vivo estimation of SERS phantom lesion depth in rat tissues. a) Schematic illustration of the preparation of the rat tissue stack and TRS measurements. TRS measurements on ex vivo b) rat muscle tissues and c) rat fat tissues (*n* = 3): (left) Experimental setup image of tissue stack, (middle) normalized Raman spectra at different ***d***, and (right) the natural logarithm plot of the ratio of different Raman peak pairs versus ***d***. The laser power density was 0.21 W cm^−2^ and the integration time was 5 s. d) Depth prediction of SERS phantom lesions in ex vivo rat tissues: (left) an image of the heterogeneous tissue stack. (right) Comparison of measured and predicted depth. The mean absolute percentage error (MAPE) between prediction and measurement was calculated. For each tissue type, three batches of measurements were conducted (*n* = 3).

After demonstrating this linear relationship, we further investigated the feasibility of our ratiometric spectroscopy strategy for depth prediction in rat models. Our first step was to determine the slope of the linear model, which, as discussed above, is the difference in attenuation coefficients (Δ ***µ***) of two Raman peaks in rat tissues. Therefore, we conducted ex vivo measurements on several homogeneous rat tissues (including muscle, fat, and skin) to obtain Δ ***µ*** for each tissue (**Figure** **4**). As shown in Figure 4a, muscle, fat, and skin tissues from rats were extracted and made into slides 2–4 mm in thickness. The SERS gel tube is also used as the phantom lesion and placed under the tissue on the detection platform. In order to ensure a uniform reflective index of the tube's surrounding environments, we put a layer of porcine muscle on the platform to hold the tube (Figure 4b,c, left panel). The presence of the porcine muscle does not affect the Raman peak ratio, since it is under the tube and not included in the optical path of Raman photon propagation to the tissue surface. The SERS gel tube is further covered by different layers of rat tissue pieces. The total thickness of rat tissues on the SERS gel is defined as depth (***d***). The Raman peaks of IR‐780 (i.e., 525, 939, 1208, 1372, 1528, and 1587 cm^−1^) can be distinguished from the background interference of rat tissue (see Figure S7, Supporting Information). These peaks in total form 15 peak pairs (see Table S1, Supporting Information). The intensity ratios of these peaks change along with increasing depth, due to differences in light attenuation. For example, the Raman peaks at 525 and 1528 cm^−1^ clearly show a difference in their attenuation (Figure 4b, middle panel). We have calculated the linear relationships between the natural logarithm of peak ratios and depth, and the lines with fit coefficient R^2^ >0.9 are displayed in Figure 4b (right panel). Similar measurements were conducted on rat fat tissue (Figure 4c) and skin (Figure S6, Supporting Information). It can be seen that the attenuation in both rat fat and skin is much higher than that of rat muscle. This is due to the light scattering of lipids in fatty tissues.^[^ ^29^ ^]^ The result is consistent with our previous studies of porcine tissues.^[^ ^21^ ^]^ The above experiments not only confirm the linear relationship between the natural logarithm of peak‐pair‐ratio and depth but also provide the Δ ***µ*** values for each tissue (summarized in Table S1, Supporting Information), which is crucial for depth prediction using ratiometric Raman spectroscopy.

With the Δ ***µ*** values known, we proceeded to demonstrate depth estimation of SERS phantom lesions in ex vivo rat tissues. At this stage, we still lack another key parameter, the intercept of the linear model, which can be calculated as the natural logarithm of the peak ratios when the depth is zero. Therefore, we directly measured the Raman spectrum of the SERS gels, and determined the natural logarithm of the peak ratios from the intercepts (data shown in Table S1, Supporting Information). Lesion depth prediction in both homogeneous and heterogeneous tissues can be achieved by selecting multiple peak‐pairs to establish an overdetermined system (Figure S5, Supporting Information). For a more accurate prediction, we do not use all 15 peak pairs. Instead, only the peak pairs with relatively higher Raman shifts and with their fitting R^2^ of over 0.9 for all three types of tissues are chosen. The selected 7 peak pairs are 525/1528, 525/1587, 939/1208, 939/1372, 939/1528, 939/1587, 1208/1528 cm^−1^ (Table S1, Supporting Information). It should be noted that the peaks at 1528 or 1587 cm^−1^ are not strong, but they have relatively large Raman shifts, leading to a higher difference of effective attenuation coefficients (i.e., Δ ***µ***) for a peak pair. This could be beneficial to improve the fitting accuracy. In our ex vivo study, we ensured that all the Raman spectra exhibited an adequate signal‐to‐noise ratio (SNR) of over 3 for the related peaks, by using a relatively long integration time of 5 s.

Depth prediction was first demonstrated on homogeneous rat muscle (Figure 4d, green columns). Muscle tissue stacks of thicknesses 0.5–2 cm were placed on top of the SERS gel tube. We performed transmission Raman measurements and calculated the intensity ratios of the above seven peak pairs. Our homogeneous model thus has seven equations with only one unknown variable (i.e., depth ***d***) (see the calculation process in Figure S8, Supporting Information). In this overdetermined system, the ***d*** value can be estimated using all the information from the Δ ***µ*** and intercepts of all seven peak pairs. The thickness of the tissue stack was measured with a vernier caliper, giving us our experimental depth measurement. The calculated depth estimation was found to have a relative error of no more than 12%. The measurements were repeated on three batches of muscle tissue. For each batch, we collected three spectra and calculated the averaged prediction depth and standard error. All the prediction results are presented in Table S2, Supporting Information. The *t*‐test is conducted between measured values and predicted values (Table S2, Supporting Information), and the p‐values are much larger than 0.05, indicating that there are no significant differences. The absolute prediction error of less than 1 mm for all three batches of muscle tissue. The prediction error could be possibly introduced by fluctuations of peak intensity and the background noise of the Raman spectrum. For homogeneous fat tissue, a similar calculation process was followed, and larger relative errors of up to 25% were observed (Figure 4d, yellow columns). This is due to the higher Δ ***µ*** values for fat tissues; in this case, even a small variation in the Raman peak intensity will lead to a higher error in estimating the depth.

Finally, we demonstrated depth prediction in heterogeneous rat tissues. Three batches of heterogeneous tissue stacks were measured. As shown in Figure 4d (left panel), batch #1 and #2 consist of rat muscle and fat tissues; while batch #3 are formed by muscle, fat, and skin tissues of different thicknesses. Even with batch #3, our most complicated model, we still have an overdetermined system using our heterogeneous model of seven equations with three unknown variables, corresponding to the thickness values of each of the three tissue types (see the calculation process in Figure S9, Supporting Information). Using the known Δ ****µ**** and intercepts of the seven peak pairs, we use a least‐square fitting method to find the three thickness values and the total ***d*** is then obtained by adding the three thickness values together. Our depth prediction results are surprisingly accurate, with relative errors of the total depths lower than 7%. Interestingly, if we look at the predictions for each tissue thickness, we see a combination of overestimation and underestimation, which seems to balance out when calculating the total thickness, leading to a relatively higher prediction accuracy (Table S3, Supporting Information). We also calculate the mean absolute percentage error (MAPE) between prediction and measurement for all the above assessments, and the MAPE value is 11.8%. These results suggest potential for depth estimation of in vivo real lesions in live rat models.

### 2.4 Non‐Invasive In Vivo Detection and Depth Estimation of Rat Sentinel Lymph Node

With the successful depth prediction in ex vivo tissues, we continued to investigate the feasibility of the SETRS technique for non‐invasive localization of in vivo deep lesions in rats. Here, the popliteal sentinel lymph node (SLN) of the rat was chosen as the lesion model. The SLNs are the first regional lymph nodes for primary tumor metastasis. The accurate location of SLNs is significant for an SLN biopsy to assess disease staging. We have reported SERS NPs for SLN detection, proving that these SERS NPs can accumulate at SLN and provide a sufficient operation window of several hours.^[^ ^30^ ^]^ Non‐invasive detection and depth determination of SLN for biopsy surgery aid the identification of lesions, shortening the surgical navigation process and reducing bleeding risk.^[^ ^31^ ^]^ We first conducted ultrasonic imaging on rat hind limbs to determine the relative location of the popliteal SLN (Figure S10, Supporting Information). It can be seen that the lymph node is oval‐shaped with a longitudinal diameter of 3–4 mm. It is located inside the gluteus muscles, and there is ≈1.5‐mm of skin (and subcutaneous fat tissues) and ≈3 mm of muscle tissue between the SLN and skin surface.

**Figure 5:**
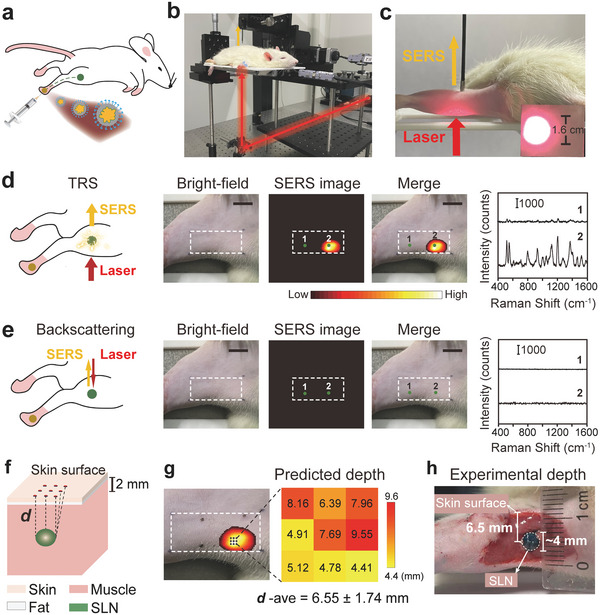
Non‐invasive in vivo detection and depth prediction of the rat popliteal SLN using SETRS within MPE. a) Schematic illustration of SERS NPs injection into the foot pad and migration to SLN. b) TRS setup and c) measurements on a rat leg to locate the lymph node. d) TRS measurements on the rat leg and the spectra collected at the corresponding marker position (*n* = 3). The laser irradiance was 0.105 J cm^−2^ and integration time was 0.5 s. e) Backscattering Raman measurements on the rat leg and corresponding spectra (*n* = 3). The laser irradiance is 1.31 J cm^−2^ and integration time was 10 ms. The bars in the bright‐field images are 1 cm for (d) and (e). Both scanning areas were of 18 × 10 mm^2^ (10 × 6 pixels) and the measurements were repeated three times for each point. f) Schematic diagram of depth prediction for SLN. g) Predicted depth and h) experimental depth of the SLN, which was stained with methyl blue dye (*n* = 3).

For the SETRS measurements of the rat popliteal SLN, the SERS NPs (100 µL, 0.5 nM) were injected subcutaneously into the foot pad of the rat, from which the NPs migrated into the popliteal SLN (**Figure** **5**a). After hair removal and disinfection of the rat leg, the rat was placed on the platform (Figure 5b), and the entire thigh was irradiated by broad beam illumination with a laser spot of ≈1.6 cm in diameter (Figure 5c). The Raman photons were collected with a fiber optic probe from the top. Wide‐area in vivo imaging was obtained by moving the fiber probe in a region of 18 × 10 mm^2^, while keeping the laser beam and rat stationary. We performed both TRS and conventional backscattering Raman measurements. In Figure 5d, a clear image of the lymph node can be observed near the tail of the inner thigh; while no characteristic Raman bands are detected from the surrounding tissue (point 1), we obtain the spectra from the lymph node positions (point 2) with Raman bands of SERS NPs clearly visible. In contrast, by using backscattering Raman spectroscopy, we were unable to detect any signal (Figure 5e). The laser energy applied for TRS is 0.105 J cm^−2^, lower than the MPE; and that for backscattering measurements is 1.31 J cm^−2^, slightly higher than the MPE threshold (see Table S4, Supporting Information).

We then demonstrated depth prediction of the popliteal SLN in vivo. In this case, the SLN is buried in a heterogeneous tissue environment consisting of skin, fat, and muscle (Figure 5f). It should be noted that, in our theoretical model, we have regarded the lesion as a “dot” without a size. However, the lymph node has a volume and the distances of its upper and lower surfaces to the skin are slightly different, so the propagation distance of Raman photons from different parts of the lymph node will vary. Therefore, the depth of SLN is not a single value but a range; and averaged depth should be regarded as the distance of the SLN center to the skin surface. We collected small‐area (3 × 3 pixels, with a 1mm step) measurements around the brightest region of TRS image (Figure 5g, left panel). This area can be regarded as the projection of the lymph node onto the skin surface. The measured 9 points were all used for depth prediction to calculate an averaged ***d*** value. Similar to the ex vivo study, we assumed that there were three types of tissue (muscle, fat, and skin), and applied a heterogeneous tissue model using the slopes of multiple peak‐pairs. For the intercepts, we determined values in advance based on the Raman spectrum of the SLN from another rat (data shown in Table S1, Supporting Information). As shown in Figure 5g (right panel), results for the 9 points give a predicted depth in the range of 4.4–9.6 mm, with an averaged ***d*** value of 6.55 ± 1.74 mm. To confirm the accuracy of this result, we directly measured the real depth of SLN (Figure 5h). Here, methylene blue dye was injected into the foot pad to stain the SLN blue. The tissues at the side edge of the leg were dissected layer‐by‐layer until the lymph node was fully exposed. The blue stain allows us to directly see the SLN with the naked eye. It can be seen that the SLN is covered by ≈1.5‐mm‐thick skin and subcutaneous fat tissue, as well as roughly 3‐mm‐thick muscle tissues, which corresponds well with the pre‐operative ultrasonic imaging. The center of the lymph node was at a depth of 6.5 mm, with a location depth range of 4.5 to 8.5 mm, matching very well with our values predicted by ratiometric TRS. To summarize, non‐invasive detection and localization of the SLN were successfully demonstrated, making it possible for us to further explore the usage of the TRS technique in practical SLN biopsy surgery.

### 2.5 Perioperative Guidance of Lymph Node Surgery Using SETRS on Rat Model

**Figure 6:**
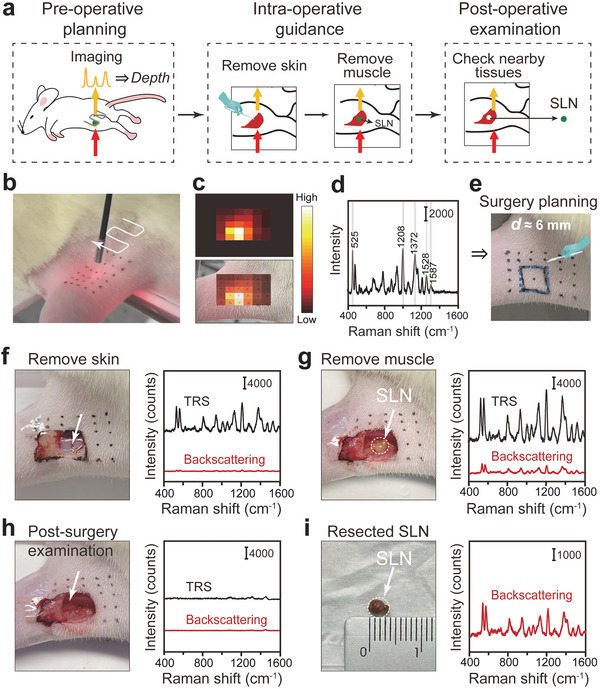
Intraoperative detection and minimal surgery of the rat SLN using SETRS. a) The schematic illustration of the perioperative surgery guidance process. b–e) Pre‐operative planning, with b) non‐invasive TRS imaging process (*n* = 3), c) corresponding TRS image and its overlay with the bright‐field image. d) Raman spectrum at the brightest spot of the TRS image. e) Pre‐operative surgical planning based on TRS imaging and depth prediction results. f–i) The TRS and backscattering Raman measurements (*n* = 3): f) after the removal of skin and subcutaneous fat, g) after the removal of muscle to expose the lymph node in situ, h) the remaining tissue after the lymph node is resected, and i) the resected lymph node. The TRS and backscattering Raman measurements were conducted with the laser irradiances of 0.105 and 1.31 J cm^−2^, and the integration time of 0.5 s and 10 ms, respectively.

Finally, we demonstrated peri‐operative guidance of SLN surgery with minimally invasive resection using TRS in live rats. Briefly, TRS can be beneficial at all stages of the clinical procedure of lymph node biopsy surgery (**Figure** **6**a): 1) pre‐operative detection of the lymph node to help plan surgery; 2) intraoperative guidance to locate the deep lymph node, and 3) post‐operative scanning of the remaining tissues to ensure no residual lesions remain.

We performed the surgery following clinical procedures. First, rapid scanning using TRS was performed on the surface of the rat leg (Figure 6b). Based on the scanning, the area of the brightest Raman intensity could be roughly determined (Figure 6c), and the corresponding Raman spectra were collected near the center of the brightest spot for depth prediction (Figure 6d). In this way, we were able to select a small area for minimally invasive open surgery; also, an estimation of SLN ***d*** (≈6 mm) was obtained to help make the surgical planning (Figure 6e). Accurate positioning of the SLN before surgery played a great role in the surgical process, allowing preliminarily determination of the thickness of tissue to be removed, to prevent over‐excision of the surrounding tissues. We then commenced surgery. Raman spectra were collected using TRS and conventional backscattering Raman spectrometry at each stage of the surgery. The skin tissue (and adjunct subcutaneous fat layer) was first removed (Figure 6f). Both the TRS signal and the backscattering Raman signal increased, indicating that we were closer to SLN. At this moment, we could not see the SLN with the naked eye, since it was still buried inside. We continued to separate the muscle tissue layer by layer, assisted by our prior knowledge of the estimated lymph node depth. We were able to cut away relatively larger pieces of tissue while the lymph node was still a distance away, then as we approached the targeted location, we could cut the tissue pieces more carefully and slowly, to avoid possible damage to the lymph node structure. After removing roughly 3‐mm muscle tissue, the whole SLN could be clearly seen, embedded in the tight gluteus (Figure S11, Supporting Information). Due to the presence of the SERS NPs inside the lymph node, it looked yellower than the surrounding tissues. After the removal of adjunct muscle tissues, the SLN was fully exposed (Figure 6g). In this case, both TRS and backscattering Raman exhibited clear and distinguishable signals from the SERS NPs. We recorded the initial cut area and the location of the exposed SLN on a glass slide, and the two regions were consistent with each other (Figure S12, Supporting Information). Also, we used a tube to measure the real depth range of the lymph node volume at this moment (Figure S13, Supporting Information); the shortest and the longest distance was about 5.1 and 8.5 mm, respectively. Therefore, the center of the lymph node (6.8 mm) was close to the pre‐operative estimation, indicating that the pre‐operative TRS provided appropriate information for surgical navigation.

Finally, the SLN was carefully resected. The adjunct tissue was examined and no characteristic Raman signals were detected by either of the two Raman modalities, demonstrating the successful resection of the lymph node with tagged NPs (Figure 6h). For double‐checking, the resected SLN was again tested using the hand‐held backscattering Raman spectrometer (Figure S14, Supporting Information); the strong signal of SERS NPs confirmed the removal of the right target (Figure 6i). The rat was anesthetized and kept alive throughout the surgery, and afterward, the resection area on the rat leg was disinfected and then sutured. We have additionally investigated the acute and the short‐term toxicity effects of NPs on rats. The rats were kept alive either for 24 hours or 10 days after the surgery, and no behavioral abnormality was observed. Finally, the rats,  were euthanized, and their key organs (heart, liver, spleen, lung, and kidney) were made into tissue sections for HE staining. No abnormalities were observed (see Figure S16, Supporting Information). However, we have to admit that the animal sample size is still small in this study, and further investigations with a larger sample size and more diverse samples are needed to validate the results.

Overall, TRS successfully assisted the whole perioperative procedure under clinically safe laser irradiation, achieving preoperative noninvasive percutaneous detection and localization. This has many advantages for surgery. The accuracy of localization using current medical imaging (CT, MRI, etc.) may be adversely affected by lesion position shift due to patient posture changes between preoperative diagnosis and intraoperative process. With our method, surgeons can determine the location immediately before surgery, improving localization accuracy. Take breast cancer SLN biopsy surgery as an example: These SLN lesions are usually distributed several centimeters below the skin and cannot be directly observed by the naked eye, even when an imaging contrast agent (e.g., blue dye) is applied. Usually, surgeons have to trace the blue dye drainage along the lymphatic vessels to locate the SLN. With the TRS technique, the location and depth of lymph nodes can be obtained percutaneously before surgery, greatly shortening the operation process. Also, foreknowledge of the lesion depth allows the doctor to determine the range of the open incision, protect the nearby blood vessels in the chest and back, and avoid over‐excision of surrounding tissues. The preoperative scanning process can be performed rapidly, taking only a few seconds for a scan with less than 0.1 s at each point. A full scan image such as in Figure 6c, does not in fact need to be produced in a real surgical scenario. Instead, the surgeons could monitor the real‐time variations of Raman intensity on the computer screen, as they move the collection probe across the animal skin surface. This allows them to move the collection probe in the direction with higher Raman intensities, rather than a zig‐zag point‐scanning for a whole square area. In the common case, only several points need to be tested to locate the range of lymph node, mostly, a few points in a line toward the lymph node, and then a few points around the lymph node (see scheme in Figure S15, Supporting Information). Once the surgeons find the projected position with the strongest signal, they could repeat the Raman measurements a few times near this position to obtain an averaged spectrum, to determine the depth of the lesion.

Finally, we would like to discuss the future directions of the TRS implementation. First, the current maximum detection depth is only a few centimeters. It is critical to improve detection capability in thicker tissues. Second, in this work, the preoperative depth prediction calculation was run using separate software. Ideally, we would like to develop software combining Raman measurements and instantaneous depth calculation. We anticipate an automated and integrated TRS scanning system for clinical use. Last, biosafety and biocompatibility remain significant. In this work we selected the lymph node as it requires a local injection, that is, the injection of NPs near the tumor. The NPs would be captured by the lymphatic system and delivered to the lymph nodes. We have reported a series of studies proving that the SERS NPs stayed and accumulated at the lymph node for several hours post‐injection.^[^ ^30^ ^]^ These NPs can be finally removed with the resection of lymph node. Compared to the intravenous injection, the delivery of NPs to the rest part of the body could be largely reduced which therefore greatly minimizes the biotoxicity. Also, we applied NPs with surface functionalization of a PEG layer with a negative zeta potential to prevent the nonspecific interactions between the proteins and NPs, weakening the SERS NPs’ interactions with the immune system and overcoming the aggregation.^[^ ^24^ ^]^ Still, we admit that the long‐term biosafety of the NPs should be investigated, which will benefit the clinical translation of our proposed SETRS techniques.

## 3 Conclusion

We have introduced the concept and demonstrated the successful application of in vivo SETRS for the non‐invasive detection, depth estimation, and peri‐operative guidance of deep SLN navigation and resectionin live rats. The SERS NPs were developed as contrast agents with a low detection limit of 10 pM. Combined with our home‐built photosafe TRS setup with a safe laser irradiance, we demonstrated the deep detection capability on 3‐cm‐thick ex vivo porcine tissues. Then, by using the ratiometric Raman spectroscopy method for lesion depth prediction, we successfully estimated the depth of SERS phantom lesion in heterogeneous tissues with high accuracy (a mean relative error of 11.8%). We further achieved non‐invasive in vivo detection and accurate localization of a 6‐mm‐deep lymph node in live rats . This further unveils the possibility of using TRS for surgery guidance of lymph node biopsy. Finally, we demonstrated the feasibility of TRS to guide all stages of surgery, including preoperative planning, intraoperative guidance, and postoperative examination, under clinically safe laser irradiation (0.21 W cm^−2^). This study provides new insights into the localization of deep lesions with significance for both fundamental studies and clinical applications.

To the best of our knowledge, this study is the first report demonstrating TRS imaging of lesions and peri‐operative surgical guidance on a live animal model. Compared to the study model (euthanatized animal model or ex vivo tissues) in the current state‐of‐the‐art reports on TRS, our studies in live animal models are quite different and more challenging, regarding the laser irradiance, injection dose, and tissue properties. For example, blood flow in living animals will aggravate the scattering of photons, resulting in lower detection depth and accuracy. Therefore, this study represents a significant step toward the real clinical transformation of TRS techniques.

## 4 Experimental Section

### Chemical and Reagents

Chloroauric chloride (HAuCl_4_·4H_2_O), ethanol (≥99.7%), *N*,*N*‐dimethylformamide (DMF, ≥99%) and agarose were received from Sinopharm Chemical Reagent Co. Ltd. (Shanghai, China). Cetyltrimethylammonium chloride (CTAC, 99%) and sodium borohydride (NaBH_4_, 98%) were purchased from J&K Chemical Ltd. (Shanghai, China). Silver nitrate (AgNO_3_, 99.8%) and ascorbic acid (>99.0%) were obtained from Aladdin (China). 4‐Nitrobenzenethiol (4‐NBT) and IR‐780 iodide (IR780, 98%) were acquired from Sigma Aldrich (Shanghai, China). Thiolated PEG (M.W. = 5000) and carboxyl PEG thiol (M.W. = 5000) were purchased from Lingfeng Ltd. (Shanghai, China). Poly(vinylpolypyrrolidone) (PVP) was purchased from Hubei Gedian Human Wellbeing Pharmaceutical Co. (China). Nanopure water (18.2 MΩ) was used for all experiments. All materials were used as received without any further purification.

### Preparation and Surface Modification of SERS NPs

SERS NPs were fabricated in two steps according to the reported protocols.^[^ ^14^, ^32^ ^]^ First, the petal‐like Au NPs (≈40–50 nm) were synthesized using a seed‐mediated method, directedly from the Au seeds of 3–5 nm. Typically, seed solution was first prepared by vigorous mixing of 4.75 mL of aqueous CTAC solution (0.1 m), 14.7 mL of water, and 258 µL of HAuCl_4_ (4.86 mm) with 224 µL of NaBH_4_ solution (0.02 m). The seed solution was held at 30 °C for 1 h in a hot bath. Then Au seeds (100 µL) were mixed with CTAC solution (0.1 m, 200 mL) and 4‐NBT molecules (4 mm, 1.4 mL) for 10 min. The 4‐NBT molecules adsorb onto the Au seed surface, and in this case, without the wash process, the modified Au seeds solution was mixed with 15 mL of HAuCl_4_ (4.86 mm) and 15 mL of ascorbic acid (0.04 m). The Au petal‐like NPs were obtained in this step. After 15 min of the reaction, the NP colloids were centrifuged to wash them three times and concentrated to 80 mL.

For the modification of Raman reporters and the growth of the Ag shell, the Au petal‐like NPs obtained in the above step (5 mL) were served as cores and mixed with 2.5 mL of IR‐780 Raman reporter molecules (1.28 mm, dissolved in DMF). After 2 h, the mixture was centrifuged to remove excess IR‐780 molecules and dispersed in 100 mL CTAC (25 mm). Then, 6.144 mL of AgNO_3_ (15 mm) and 10 mL of ascorbic acid (800 mm) were added to the above molecule‐modified Au NPs and the mixture was incubated for 2 h at 70 °C to form the Au/Ag bimetallic SERS NPs. After washing and re‐dispersion in an aqueous solution, the final concentration of SERS NPs was dispersed in water. The concentration was determined by nanoparticle tracking analysis (Zetaview x30, Particle Metrix), and finally, the nanoparticles were concentrated to 0.5 nm.

For the surface modification of SERS NPs, 1 mL of the as‐synthesized Au/Ag GERRT NPs was taken and 500 µL of carboxyl PEG thiol aqueous solution (259 *µ* m) was added. After incubation for 30 min, another 800 µL of thiol PEG aqueous solution (259 *µ* m) was added. The mixture was allowed to shake for another 1 h for incubation to ensure maximum coating of PEG molecules onto the Au surface. After that, the mixture was centrifuged and washed with water thoroughly to remove excess CTAC and PEG molecules. Finally, the NPs were dispersed in 1% PVP solution.

The morphologies of the SERS NPs were confirmed using TEM images, collected from a TEM operated at 200 kV (JEM‐2100F, JEOL, Japan). The UV–vis extinction spectra of the NPs were measured with a UV–vis spectrophotometer (UV1900, Aucybest, China). The hydrodynamic size and zeta potential were tested on a Zetasizer instrument (NanoZS, Malvern, U.K.). The Raman characterization of NPs was performed using the i‐Raman portable spectrometer (B&WTEK, U.S.).

### Transmission Raman Spectroscopy Setup

For the transmission Raman setup, a miniaturized Raman fiber optic probe (EMvision HT‐PROB‐ENDO‐785 Raman probe) was used as the collection probe, which was connected to a Raman spectrometer (Andor Ivac‐316 CCD). The bundle probe consists of six fibers arranged in a ring‐shape with the diameter of each fiber as 300 µm.^[^ ^33^ ^]^ The sample was placed on a sample stage. The collection probe was put on an automatic XY moving stage to collect Raman signal from the top of the sample. The Raman spectrometer (i‐Raman, BWTEK, USA) with a hand‐held probe was used as the laser source. The lens of the laser probe was removed to produce the broad‐beam laser with a maximum laser power of 420 mW. The laser beam was incident on the bottom surface of the sample with a spot of ≈0.8 cm in radius, which leads to a low power density (≈0.21 W cm^−2^). To form the transmission Raman setup, the laser illumination, and Raman signal collection were on opposite sides of the tissues (Figure S1, Supporting Information).

### Ex Vivo Experiments on Porcine Tissues

The ex vivo studies for TRS detection were performed using SERS gels as lesion phantoms. To prepare the SERS gels, 5 wt% agarose solution was obtained by heating the mixture of agarose and water in a microwave oven until fully dissolved, and then mixed with SERS NPs to obtain a final NP concentration of 0.5 nm. The mixture was then stirred to ensure the SERS NP colloids were well‐dispersed in the agarose solution. The solution was filled into a small tube and cooled down to form the gel. The SERS gel tube was with a length of 5 mm and an inner diameter of 1.5 mm (Figure S2, Supporting Information).

The ex vivo porcine muscle tissues were purchased fresh from a local market. They were frozen before use and dissected into slices by an electric meat slicer (SL‐518, Chigo, China) with a thickness of 0.3 cm. Considering the softness of tissue samples, a deviation of 10–15% in tissue thickness was acceptable. The length and width of the slices were both more than 4 cm. The porcine muscle tissue stack was 3 cm in thickness, with 10 layers of tissues. For the ex vivo TRS measurements, the SERS gel tube was embedded in the tissue stacks with different buried depths. The depths were adjusted by changing the position of the SERS gel tube along the optical axis (*z*–axis) within the tissue slabs. The power density of 0.21 W cm^−2^ and the integration time of 5 s were applied.

### Ex Vivo Depth Prediction on Rat Tissues

The depth prediction models were based on the Δ ****µ**** values of the Raman peak pairs. To measure these values, rat muscle/fat/skin tissues were first obtained. The slices of rat muscle/fat tissue were resected from the legs of a euthanatized rat. These slides were of 2–4 mm thickness. Rat skin tissue was obtained by peeling off the skin from this euthanatized rat body. A 4‐mm‐thick layer of porcine muscle was placed in the holder as a substrate, with the prepared SERS gel placed in the center of the porcine muscle. This was to ensure a uniform reflective index of the surrounding environments around the SERS gel tube. Then, 5–6 layers of rat tissues were placed on top of the SERS gel one by one, with the thickness of each slice measured by a vernier caliper. The transmission Raman measurements were conducted after each new layer of rat tissue was placed. The measurements were repeated three times for each data point to calculate an averaged peak intensity. The power density of 0.21 W cm^−2^ and the integration time of 5 s were applied. Six Raman peaks of IR‐780 at 525, 939, 1208, 1372, 1528, and 1587 cm^−1^ were selected. The natural logarithm of intensity ratios was calculated, and their relationship with thicknesses (i.e., depth, ***d***) was obtained. In this way, the linear models for depth prediction were obtained. The slope (i.e., Δ ****µ****) and the intercept were obtained from this linear model. 7 peak pairs (525/1528, 525/1587, 939/1208, 939/1372, 939/1528, 939/1587, 1208/1528) were further selected for further depth prediction. For each tissue type, three batches of measurements were conducted (*n* = 3) and the measurements were repeated three times for each batch.

For the depth prediction on homogeneous rat tissues, the linear relationship was obtained for each Raman peak pair (Figure S4, Supporting Information). An overdetermined system was defined by these seven linear models with an unknown ***d***. The least‐square solution for ***d*** in the overdetermined equation was obtained. As for the depth prediction in heterogenous tissue consisting of muscle and fat, a more complex relationship of intensity ratios and depth was modeled. Each Raman peak pair was in possession of two Δ ****µ**** values (one for the muscle tissue and the other for the fat tissue) and one invariant intercept determined only by the Raman spectrum of SERS NPs. The unknown depth ***d*** was composed of thicknesses of muscle (***d***_***muscle***_) and fat (***d***_***fat***_). Therefore, a piecewise linear relationship was obtained for each Raman peak pair, where a change of slope existed in the boundary of two homogeneous tissues (Figure S5, Supporting Information). Similarly, an overdetermined system was defined by seven equations with two unknown depth values. The least‐square solution was obtained for the thickness of each tissue and a total ***d*** could be calculated. In heterogeneous tissue consisting of muscle, fat, and skin, a similar model was utilized with three Δ ****µ**** values for each Raman peak pair. For heterogeneous tissues, three batches of measurements were conducted (*n* = 3) and the measurements were repeated three times for each batch.

### Non‐Invasive In Vivo Detection and Depth Prediction of the Lymph Node on the Rat Model

Animal experiments were approved by the Institutional Animal Care and Use Committee at Shanghai Jiao Tong University (A2020019 ), and the Bioethics Committee of School of Biomedical Engineering (no. 2021007). All animal housing and experiments were conducted in accordance with the ethical standards of the institute.

The rats (male Sprague Dawley rat model, 6–8 weeks) were purchased from Shanghai Jiesijie Laboratory Animal Co., LTD (Shanghai, China). The hair on the hind legs of the rats was shaved. The rats were anesthetized with an intraperitoneal injection of 1.5–2.5 mL sodium pentobarbital (1%). The SERS NP colloids, consisting of 100 µL of 0.5 nM NPs dispersed in a 1% PVP saline solution, were then injected subcutaneously into the foot pads of the rats left hind leg. 3 h after injecting the SERS NPs, the rats were anesthetized again using 1% sodium pentobarbital and placed on a TRS detection platform. The optical path was adjusted so that the thigh of the rat, laser, and the receiving probe were aligned vertically, with the laser spot size (0.8 cm in radius) illuminating the skin surface of the thigh from the bottom. The excitation laser beam was then fixed and the receiving probe could be moved by an automatic *XY*‐translational stage.

As the rough location of the sentinel lymph node was known from the ultrasonic image (Figure S10, Supporting Information), the nearby surface skin was marked and a small scanning range was performed in that area. The scanning area was 18 × 10 mm^2^ with a step length of 2 mm, for a total of 60 points. For the TRS scanning, the power density of 0.21 W cm^−2^ and the integration time of 0.5 s were applied, corresponding to the laser irradiance of 0.105 J cm^−2^. For the conventional backscattering Raman setup, a portable Raman spectrometer (i‐Raman, BWTEK, U.S.) was applied with a focusing lens at the end of the laser probe. The 785 nm laser beam was incident on the tissue surface with the integration time of 10 ms, and the laser power at the tissue surface of 10 mW (131 W cm^−2^, with a focal spot of ≈100 µm in diameter, corresponded to 1.31 J cm^−2^ with 10‐ms integration time). The laser irradiance was slightly higher than the MPE threshold (0.51 J cm^−2^) with the same integration time. At each point, the measurements were repeated three times to get an averaged value for both TRS and backscattering Raman modalities. The rat remained alive after all measurements. The Raman images were plotted using the peak intensity at 525 cm^−1^. All SERS images were smoothened by an interpolation algorithm. For SERS pseudo‐color mapping, the threshold was set as 1/e of the spot center, that is, for the points whose intensity was lower than the threshold were plotted as black.

For depth estimation of the lymph node, the data of small‐region TRS mapping was used. The brightest spot in the large‐region TRS image can be regarded as the projection point of the lymph node onto the skin surface. Small‐area TRS mapping was then performed over an area of 3 mm × 3 mm (3 × 3 pixels) around the brightest region. This region was not exactly at the center of theSLN image. The measured 9 points were used for the depth prediction of this lymph node. For the prediction, it was assumed that there were either three types of tissues (muscle, fat, and skin) or one type of tissue (muscle, as it was the main tissue on the rat leg). The intercept values were measured in advance before this experiment: the SERS NPs were injected onto the footpad of another rat; and at 3 h after injection, its SLN was taken and the Raman spectra were tested. The intercepts were obtained based on the averaged Raman spectrum. Within known intercepts and slopes (i.e., the attenuation coefficients), the same calculation processes as the assessments on ex vivo tissues were conducted here. The predicted depths of the 9 points were plotted in a grid view color map.

To compare the predicted depth and the real depth of the lymph node, 50 µL of methylene blue dye was injected at the foot pad to stain the lymph node blue. After 5‐min post‐injection, the tissues at the side surface of the SLN were dissected layer‐by‐layer, until the lymph node was fully exposed. The depth of SLN was measured using a ruler.

### In Vivo Perioperative Guidance of Lymph Node Biopsy Surgery on the Rat Model

Perioperative guidance includes pre‐, intra‐, and post‐operative imaging and guidance during the surgery. The rat was treated in the same way as the non‐invasive detection of rat lymph nodes. As for the intraoperative guidance, non‐invasive TRS mapping was conducted to get preoperative positioning information on lymph node location. The brightest spot, that is, the projection point of the lymph node onto the skin surface, was determined. The spectrum at the brightest spot was extracted and analyzed for depth prediction. A minimal surgery region was determined around the brightest area. Based on the information, the skin and muscle were then removed step‐by‐step to expose the lymph node. For each step of the intra‐operative process, both TRS and the backscattering Raman measurements were used and recorded as a comparison. For the conventional backscattering Raman measurements, the Raman spectrometer (i‐Raman, BWTEK) was applied with a focusing lens at the laser exit of its hand‐held probe. The laser irradiance power density of both TRS and backscattering Raman spectroscopy is shown in Table S4, Supporting Information.

### Raman Data Analysis

The background of raw Raman spectra was corrected using the adaptive iteratively reweighted Penalized Least Squares (airPLS) algorithm to remove the tissue autofluorescence background while preserving characteristic Raman peaks. The peak intensities were calculated in the following way: the peak intensity (*I_peak_*)was recorded as the maximum value of each Raman band, and the baseline floor (*I_baseline_*) was calculated as the mean value in the region of 1800–1850 cm^−1^, where there were no specific Raman signals. Therefore, the final intensity of a Raman band was calculated as follows: *I*  = *I*_peak_  − *I*_baseline_. To ensure the calculation accuracy, only the Raman spectrum was applied, whose peaks (i.e., 525, 939, 1208, 1372, 1528, and 1587 cm^−1^) had an SNR of over 3, for the depth prediction calculation. Spectra with lower SNR would be eliminated. The spectral SNR was calculated by diving the peak intensity and the standard deviations of data at 1800–1850 cm^−1^ (i.e., as noise).

### Statistical Analysis

1) Pre‐processing of data: The background of raw Raman spectra was corrected using the airPLS. 2) Data presentation: The data measured or derived from experiments in this study were presented as mean ± SD. 3) Sample size for each statistical analysis: a) For Figure 4b,c measurements of Δ*µ* on each type of rat tissues were repeated (*n* = 3), and every data point was tested three times. b) For Figure 4d, each tissue type (homogeneous muscle, homogeneous fat, and heterogeneous tissues) was repeatedly tested (*n* = 3), and each thickness was tested repeatedly three times. c) Figure 5 (*n* = 3). d) Figure 6 (*n* = 3). 4) One sample Student's *t*‐test (two sides, the significance level was 0.05) was used to assess significant differences between measured values and predicted values of depths (for Figure 4) in Tables S2 and S3, Supporting Information. 5) Software used for statistical analysis: All the statistical analyses were conducted in Origin (2021) and MATLAB (2019).

## Conflict of Interest

The authors declare no conflict of interest.

## Author Contributions

Z.W. contributed to methodology, formal analysis, investigation, data curation, writing—original draft, and visualization. B.D. contributed to supervision, methodology, investigation, visualization, and writing—review and editing. Yut.Z. contributed to investigation and visualization. H.X. contributed to formal analysis, validation, investigation, and data curation. Yum.Z. contributed to methodology. L.L. contributed to supervision, resources, validation, visualization, funding acquisition, and writing—review and editing. J.Y. contributed to administration, validation, resources, funding acquisition, and writing—review and editing. All authors have read and agreed to the published version of the manuscript.

## Supporting information

Supporting Information

## Data Availability

The data that support the findings of this study are available from the corresponding author upon reasonable request.
