# Supplementary material for: Non‐Invasive Detection, Precise Localization, and Perioperative Navigation of In Vivo Deep Lesions Using Transmission Raman Spectroscopy
Source: Adv Sci (Weinh). 2023 Jun 20;10(24):2301721. doi: 10.1002/advs.202301721 (PMC10460859; doi:10.1002/advs.202301721)
Supplement: Supplementary file 1 — Supporting Information [file ADVS-10-2301721-s001.pdf]

ADVANCED SCIENCE
Open Access

Supporting Information

for *Adv. Sci.*, DOI 10.1002/advs.202301721

Non-Invasive Detection, Precise Localization, and Perioperative Navigation of In Vivo Deep Lesions Using Transmission Raman Spectroscopy

Zongyu Wu, Binge Deng, Yutong Zhou, Haoqiang Xie, Yumin Zhang, Li Lin* and Jian Ye**

Non-invasive detection, precise localization, and perioperative navigation of *in vivo* deep lesions using transmission Raman spectroscopy

Zongyu Wu[1], Binge Deng[1]*, Yutong Zhou[1], Haoqiang Xie[1], Yumin Zhang[1], Li Lin[1]*, Jian Ye[1,2,3]*

1 State Key Laboratory of Systems Medicine for Cancer, School of biomedical engineering, Shanghai Jiao Tong University, Shanghai 200030, P. R. China

2 Institute of Medical Robotics, Shanghai Jiao Tong University, Shanghai 200240, P.R. China

3 Shanghai Key Laboratory of Gynecologic Oncology, Ren Ji Hospital, School of Medicine, Shanghai Jiao Tong University, Shanghai 200127, P. R. China

* Correspondence: bingo_dn@sjtu.edu.cn (B.D.); linli92@sjtu.edu.cn (L.L.); yejian78@sjtu.edu.cn (J.Y.)

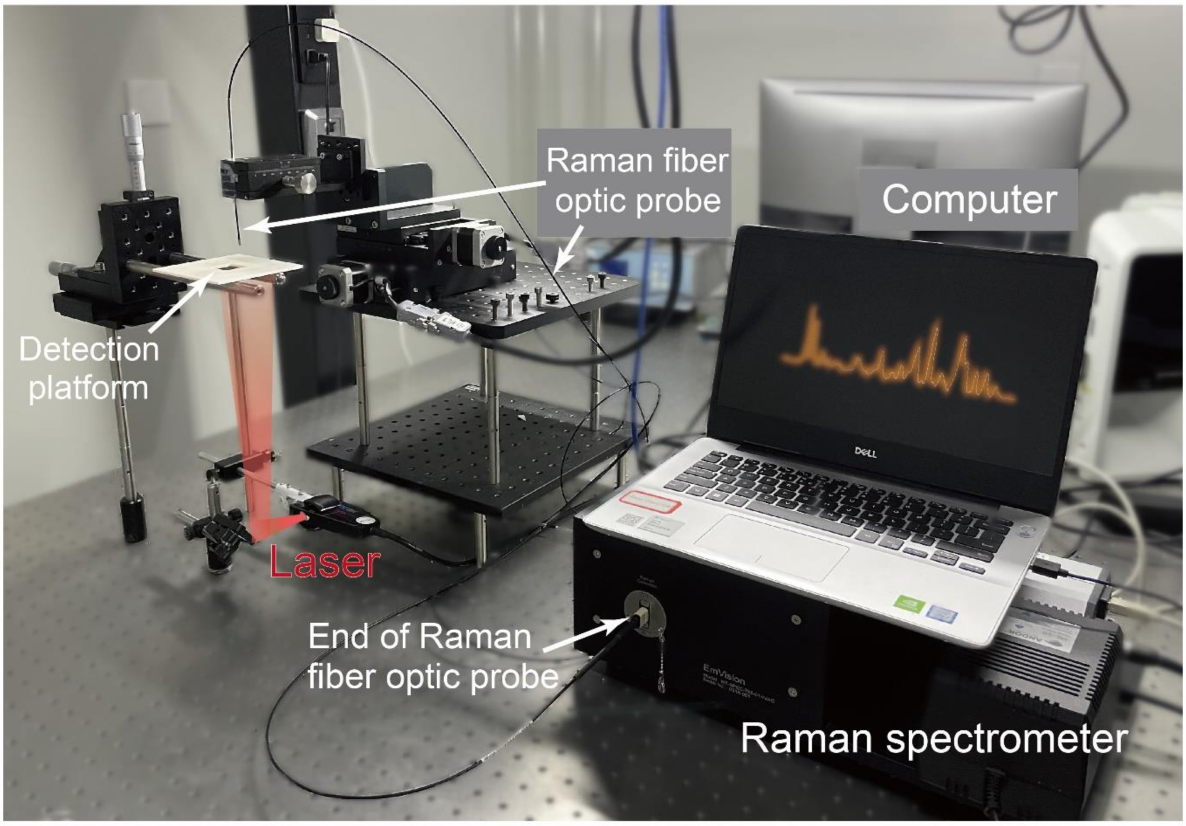

Figure S1. The experimental transmission Raman spectroscopy (TRS) setup. The excitation laser of 785 nm is emitted from the laser probe of a Raman spectrometer (i-Raman, BWTEK, USA), then is reflected by a NIR reflecting mirror, passing through a hole (2.5 cm * 2.5 cm) of the detection platform, and finally illuminate on the bottom of the sample placed on the platform. Emitted Raman photons are collected by the fiber bundle probe (EMvision HT-PROB-ENDO-785 Raman probe). The fiber optic is connected to a Raman spectrometer (Andor Ivac-316 CCD). Finally, Raman spectra are presented on a computer. The maximum laser power is 420 mW, and the laser spot size could be well adjusted in the range of 0.8 – 1.7 cm, by controlling the distance between the laser probe to the sample stage. In this work, we apply a laser spot size of 1.6 cm, which leads to a power density of 0.21 W/cm^2.

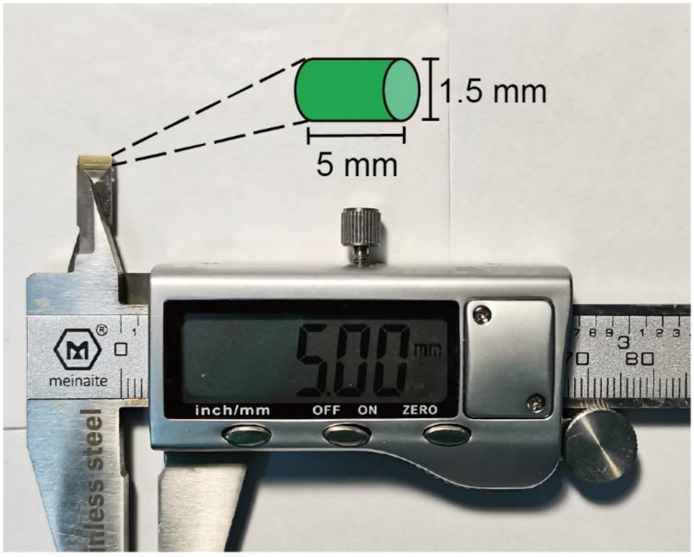

Figure S2. The size of SERS gel tube. The length of the SERS tube is 5.00 mm and the inner diameter of the tube is 1.50 mm.

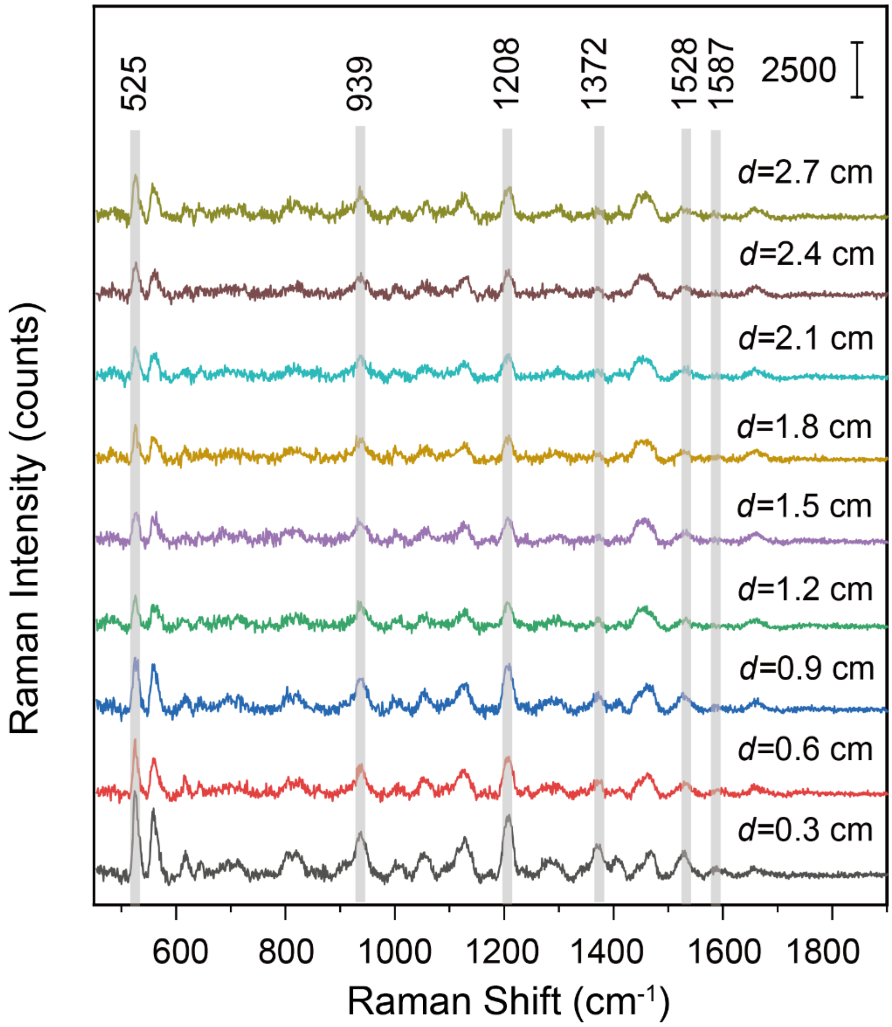

Figure S3. All the Raman spectra of SETRS tests on porcine muscle with varying depths. The spectra data were the same to Figure 3d. The laser power density was 0.21 W/cm^2 and the integration time was 5 s.

The "ratiometric Raman spectroscopy" method for depth estimation of lesions

Here we theoretically demonstrate the ratiometric Raman spectroscopy method. The photon propagation in the homogeneous tissue can be described using the Radiative Transfer Equation (RTE). First, we regard the lesion tagged by SERS nanotags as a point source generating Raman photons. The propagation of Raman photons through the tissue can be modeled using the steady-state diffusion approximation:

$$I = I_0 \frac{e^{-\mu r}}{4\pi D r} \quad (1)$$

Where I_0 is the original Raman intensity emitting by the lesion, μ represents the effective attenuation coefficient, D represents the photon diffusion coefficient of Raman photons and the distance between (x, y, z) and the SERS nanotags-labeled lesion is r.

We defined d as the lesion depth, i.e., the distance between the SERS-labeled lesion and the nearest point at the skin surface. The Raman intensity detected at the point could be described as the following formula:

$$I_{\lambda i} = I_0 \frac{e^{-\mu(\lambda_\mathrm{i})d}}{4\pi D(\lambda_\mathrm{i})d} \quad (2)$$

where I_0 is the initial SERS intensity, $D(\lambda_i)$ is the photon diffusion coefficient, and $\mu(\lambda_i)$ is the effective attenuation coefficient of the tissue, which could be calculated from the absorption coefficient μ_a and the reduced scattering coefficient μ_s'. λ_i is the wavelength of emission Raman photons (known). Here in two Raman peaks are selected, with their wavelengths of λ_1 and λ_2, respectively, the difference between the effective attenuation coefficients at the two wavelengths is:

$$\Delta\boldsymbol{\mu} = \mu(\lambda_2) - \mu(\lambda_1) \quad (3)$$

In combination with Equations (2) and (3), the natural logarithm of the intensity ratio of two Raman peaks in detected Raman spectrum is :

$$\ln\left(\frac{I_{\lambda 1}}{I_{\lambda 2}}\right) = \Delta\mu \ \cdot \boldsymbol{d} + \ln\left(\frac{D(\lambda_2)}{D(\lambda_1)}\right) \quad (4),$$

This indicates a linear relationship between the depth ($\boldsymbol{d}$) and the natural logarithm of the peak ratio, i.e., $\ln\left(\frac{I_{\lambda 1}}{I_{\lambda 2}}\right)$. And the slope of the line presents the $\Delta\boldsymbol{\mu}$ of the corresponding Raman peak pair in this kind of tissue (**Figure S4a**). The linear relationship provides the possibility for lesion depth estimation.

To avoid possible estimation errors using only one peak pair, the combination of multiple peak pairs is preferred. Raman spectrum exhibits multiple characteristic peaks, and the linear relationship between the natural logarithm of peak intensity-ratio and depth could be found for different Raman peak pairs. Therefore, we select multiple pairs of Raman peaks to establish the linear relation model. Then, by combining all linear models of these Raman peak pairs, a determined system of equations can be constructed to solve the unknown value of depth in a homogeneous tissue (**Figure S4b**). More peak pairs could be added in to improve the accuracy of depth prediction.

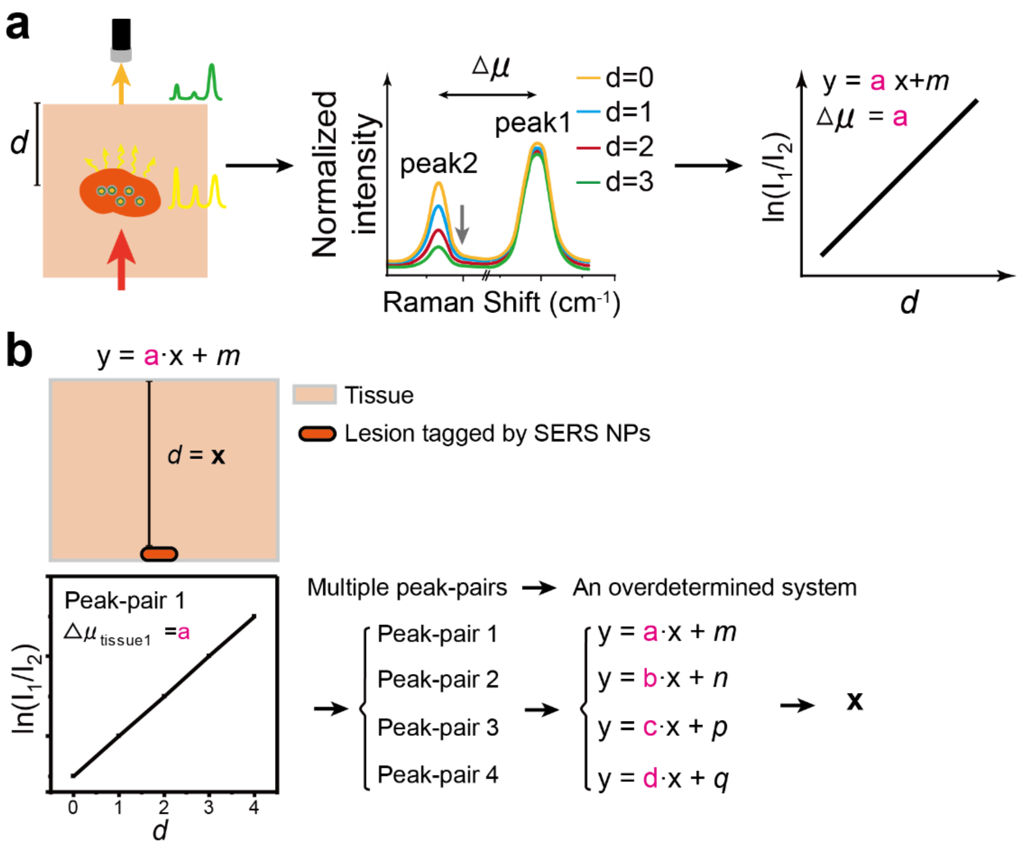

Figure S4. Schematic illustration of ratiometric Raman spectroscopy for depth prediction in homogeneous tissues. (a) The derivation for the linear model between the depth (***d***) and the natural logarithm of the peak ratio, i.e., $\ln\left(\frac{I_{\lambda 1}}{I_{\lambda 2}}\right)$. (b) The demonstration of depth prediction based on the linear model.

Similarly, the heterogeneous tissues can be observed as a stack consisting of several types of homogenous tissues. As shown in **Figure S5**, Raman photons need to pass through three types of tissues to reach the collection surface, and ***d*** is the sum of tissues of all three tissues. Since the $\Delta\boldsymbol{\mu}$ value is different for each tissue type, in this case, a broken-line relationship with two turn points will be observed, and the ***d*** is the superposition of linear models for each homogeneous tissue. There are three unknown variables: X_1, X_2, and X_3. In this case, we should select at least 3 pairs of Raman peaks to establish a determined system of equations, to solve all the unknown values of X1, X2, and X3; this is to get the final depth in a heterogeneous tissue. In this model, the total thickness of each type of tissue would be not influenced by their relative position on the SERS gel; let's say, if there are two layers of muscle tissues on the lesion, no matter if they are adjunct or separated by another tissue, the total

predicted thickness of muscle tissue stays the same. More peak pairs could also be added to form an overdetermined equation system, to improve the accuracy of depth estimation.

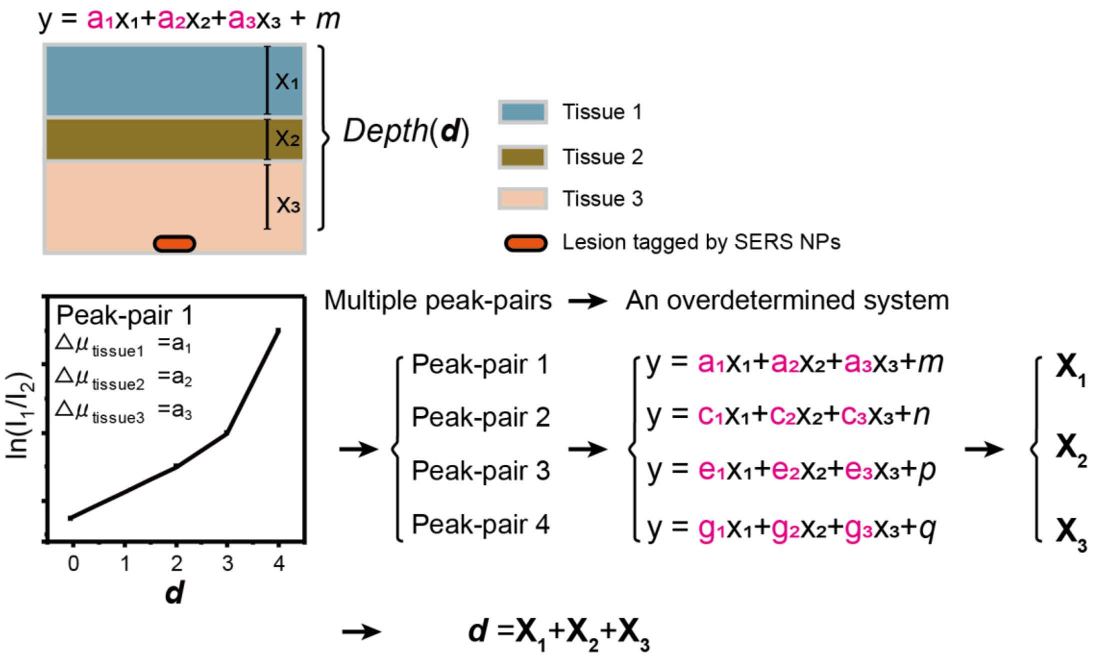

Figure S5. Schematic illustration of ratiometric Raman spectroscopy for depth prediction in heterogeneous tissues. A broken-line relationship with two turn points will be obtained here. An overdetermined system similar to that in homogeneous tissues is established to obtain the prediction depth.

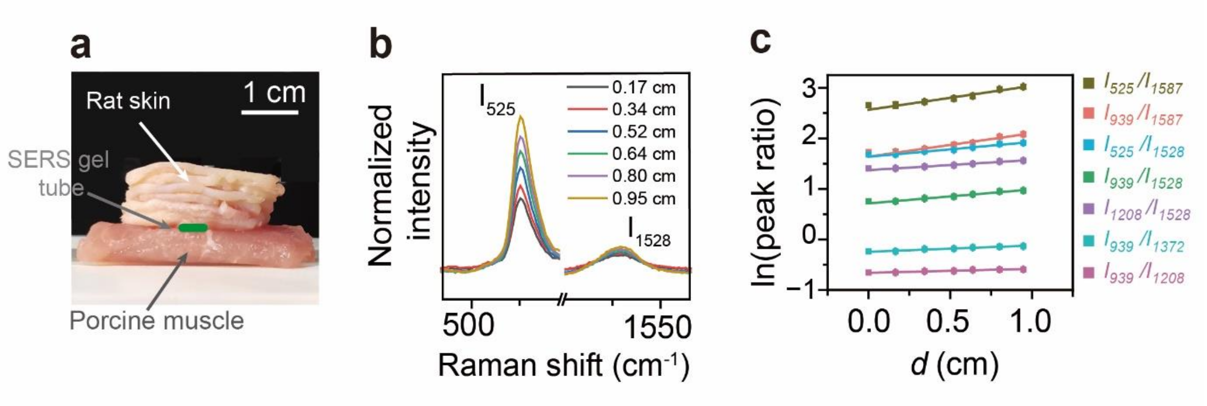

Figure S6. The transmission Raman tests demonstrated on rat skin tissues (n=3). (a) The picture of tissue stack formed of rat skin, with the SERS gel tube placed on the bottom of skin tissues. (b) The normalized Raman spectra for different depth of buried SERS gel tube. (c) The profile of depth versus the natural logarithm values of peak ratios.

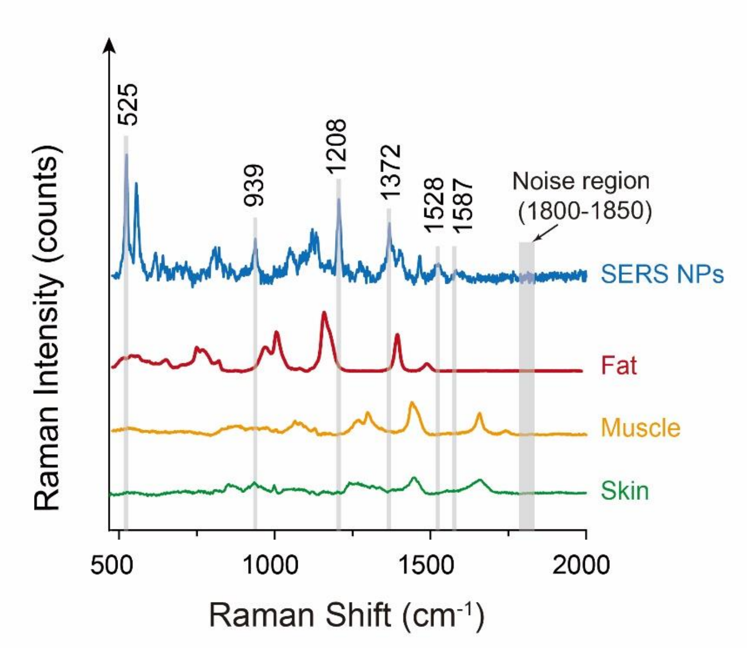

Figure S7. Raman spectra of SERS NPs (50 pM) and the rat tissue (fat, muscle and skin). These Raman spectra are baseline-subtracted. The characteristics peaks of NPs at 525, 939, 1208, 1372, 1528 and 1587 cm^{-1} can be well distinguished from that of the rat tissues. There are no distinct Raman peaks in the range of 1800-1850 cm^{-1}; we therefore use data in this region to calculate the deviation, which is regarded to present the noise levels of the spectrum.

Table S1. The $\Delta\boldsymbol{\mu}$ and linear fitting R^2 values of total 15 Raman peak pairs measured on *ex vivo* rat tissues. The intercepts measured for both *ex vivo* lesion phantom and *in vivo* SLN are also presented.

Raman peaks (cm^{-1})		*Muscle*		*Fat*		*Skin*		*Intercept*	
		$\Delta\boldsymbol{\mu}$	R^2	$\Delta\boldsymbol{\mu}$	R^2	$\Delta\boldsymbol{\mu}$	R^2	*Ex vivo*	*In vivo*
525	939	-0.114	95.2%	0.087	88.6%	0.140	87.2%	0.960	0.534
525	1208	0.029	89.0%	0.192	90.7%	0.096	93.3%	0.236	-0.156
525	1372	-0.010	90.1%	0.271	96.7%	0.137	93.7%	0.671	-0.026
*525	1528	0.114	98.3%	0.371	99.0%	0.294	97.8%	1.624	0.809
*525	1587	0.198	94.5%	0.390	99.3%	0.474	97.6%	2.638	1.603
*939	1208	0.124	95.8%	0.221	99.5%	0.078	92.4%	-0.689	-0.689
*939	1372	0.128	98.8%	0.299	99.1%	0.126	94.0%	-0.239	-0.560
*939	1528	0.203	96.2%	0.413	99.3%	0.277	99.1%	0.633	0.276
*939	1587	0.298	96.3%	0.416	95.8%	0.463	98.4%	1.641	1.069
1208	1372	-0.022	86.3%	0.080	93.9%	0.047	90.9%	0.431	0.129
*1208	1528	0.078	92.0%	0.175	95.9%	0.203	99.2%	1.393	0.965
1208	1587	0.170	92.9%	0.137	80%	0.380	96.6%	2.385	1.759
1372	1528	0.111	97.2%	0.119	93.2%	0.157	97.6%	0.948	0.836
1372	1587	0.172	90.4%	0.210	87.5%	0.335	95.1%	1.960	1.629
1528	1587	0.843	91.7%	-0.136	85.6%	0.191	92.0%	0.634	0.794

The total 15 peak pairs are calculated.

*We selected 7 peak pairs that are: (1) with R^2 of over 90% for all three types of tissues, (2) with relatively higher Raman shifts. These 7 peak pairs (marked by *) are applied for the depth prediction.

$$\begin{bmatrix} \ln(I_{525}/I_{1528}) \\ \ln(I_{525}/I_{1587}) \\ \ln(I_{939}/I_{1208}) \\ \ln(I_{939}/I_{1372}) \\ \ln(I_{939}/I_{1528}) \\ \ln(I_{939}/I_{1587}) \\ \ln(I_{1208}/I_{1528}) \end{bmatrix} = \begin{bmatrix} \Delta\mu_{muscle1} \\ \Delta\mu_{muscle2} \\ \Delta\mu_{muscle3} \\ \Delta\mu_{muscle4} \\ \Delta\mu_{muscle5} \\ \Delta\mu_{muscle6} \\ \Delta\mu_{muscle7} \end{bmatrix} [\mathbf{X}] + \begin{bmatrix} b_1 \\ b_2 \\ b_3 \\ b_4 \\ b_5 \\ b_6 \\ b_7 \end{bmatrix}$$

$$\downarrow$$

$$\begin{bmatrix} 1.963 \\ 2.950 \\ -0.466 \\ -0.009 \\ 1.045 \\ 2.032 \\ 1.511 \end{bmatrix} = \begin{bmatrix} 0.114 \\ 0.198 \\ 0.124 \\ 0.128 \\ 0.203 \\ 0.298 \\ 0.078 \end{bmatrix} [\mathbf{X}] + \begin{bmatrix} 1.624 \\ 2.638 \\ -0.689 \\ -0.239 \\ 0.633 \\ 1.641 \\ 1.393 \end{bmatrix}$$

$$\downarrow$$

$$\boldsymbol{d} = \mathbf{X} = 1.668 \text{ cm}$$

Figure S8. The calculation process and results of depth prediction on *ex vivo* homogeneous muscle tissue (#2 batch). The intercepts for the prediction model are shown. A relative error ±1~3% would be obtained considering the difference in significant digit utilized in calculation.

Table S2. The results for depth prediction on *ex vivo* homogeneous rat tissues (n=3 for each tissue type). One sample student t-test (two sides, significance level is 0.05) was used to assess significant differences between measured values and predicted values of depths. P-values higher than 0.05 are obtained, presenting no significant difference between the measured values and predicted values.

	**Muscle**			**Fat**		
	**#1**	**#2**	**#3**	**#1**	**#2**	**#3**
d-measured (cm)	0.741	1.154	1.746	0.570	0.745	0.910
Stand deviation (cm)	0.042	0.061	0.052	0.047	0.063	0.048
d-predicted (cm)	0.651	1.130	1.668	0.720	0.935	1.080
Stand deviation (cm)	0.073	0.081	0.102	0.063	0.087	0.078
relative error	-12%	-2%	-4%	+25%	+25%	+18%
p value of t-test	0.166	0.659	0.316	0.054	0.063	0.064

$$\begin{bmatrix} \ln (I_{525}/I_{1528}) \\ \ln (I_{525}/I_{1587}) \\ \ln (I_{939}/I_{1208}) \\ \ln (I_{939}/I_{1372}) \\ \ln (I_{939}/I_{1528}) \\ \ln (I_{939}/I_{1587}) \\ \ln (I_{1208}/I_{1528}) \end{bmatrix} = \begin{bmatrix} \Delta\mu_{skin1} & \Delta\mu_{fat1} & \Delta\mu_{muscle1} \\ \Delta\mu_{skin2} & \Delta\mu_{fat2} & \Delta\mu_{muscle2} \\ \Delta\mu_{skin3} & \Delta\mu_{fat3} & \Delta\mu_{muscle3} \\ \Delta\mu_{skin4} & \Delta\mu_{fat4} & \Delta\mu_{muscle4} \\ \Delta\mu_{skin5} & \Delta\mu_{fat5} & \Delta\mu_{muscle5} \\ \Delta\mu_{skin6} & \Delta\mu_{fat6} & \Delta\mu_{muscle6} \\ \Delta\mu_{skin7} & \Delta\mu_{fat7} & \Delta\mu_{muscle7} \end{bmatrix} \begin{bmatrix} \mathbf{X}_1 \\ \mathbf{X}_2 \\ \mathbf{X}_3 \end{bmatrix} + \begin{bmatrix} b_1 \\ b_2 \\ b_3 \\ b_4 \\ b_5 \\ b_6 \\ b_7 \end{bmatrix}$$

$$\downarrow$$

$$\begin{bmatrix} 1.868 \\ 2.898 \\ -0.559 \\ -0.107 \\ 0.949 \\ 1.978 \\ 1.508 \end{bmatrix} = \begin{bmatrix} 0.294 & 0.371 & 0.114 \\ 0.474 & 0.390 & 0.198 \\ 0.078 & 0.221 & 0.124 \\ 0.126 & 0.299 & 0.128 \\ 0.277 & 0.397 & 0.203 \\ 0.463 & 0.416 & 0.298 \\ 0.203 & 0.175 & 0.078 \end{bmatrix} \begin{bmatrix} \mathbf{X}_1 \\ \mathbf{X}_2 \\ \mathbf{X}_3 \end{bmatrix} + \begin{bmatrix} 1.624 \\ 2.638 \\ -0.689 \\ -0.239 \\ 0.633 \\ 1.641 \\ 1.393 \end{bmatrix}$$

$$\downarrow$$

$$\begin{cases} \mathbf{X}_1 = 0.135 \text{ cm} \\ \mathbf{X}_2 = 0.383 \text{ cm} \\ \mathbf{X}_3 = 0.373 \text{ cm} \end{cases} \longrightarrow \boldsymbol{d} = \mathbf{X}_1 + \mathbf{X}_2 + \mathbf{X}_3 = 0.891 \text{ cm}$$

Figure S9. The calculation process for heterogeneous tissues (#3 batch) formed of three kinds of tissues (rat skin, fat, and muscle). A relative error ±1~3% would be obtained considering the difference in significant digit utilized in calculation.

Table S3. The results for depth prediction on *ex vivo* heterogeneous rat tissues (n=3). One sample student t-test (two sides, significance level is 0.05) was used to assess significant differences between measured values and predicted values of depths. P-values higher than 0.05 are obtained in heterogeneous tissues, presenting no significant difference between the measured values and predicted values.

	**# 1**		**# 2**		**# 3**		
	Fat	Muscle	Fat	Muscle	Skin	Fat	Muscle
d-measured (cm)	0.389	0.410	0.389	0.577	0.168	0.389	0.410
d-predicted (cm)	0.229	0.628	0.304	0.601	0.135	0.383	0.373
relative error	-40%	+53%	-22%	4%	-20%	-2%	-9%
total-*d*-measured (cm)	0.799		0.966		0.967		
stand deviation (cm)	0.039		0.061		0.063		
total-*d*-predicted (cm)	0.857		0.905		0.891		
stand deviation (cm)	0.069		0.074		0.065		
relative error	+7%		-6%		-7%		
p value of t-test	0.283		0.290		0.177		

Non-invasive ultrasonic imaging of the lymph node

The ultrasonic imaging was performed using an ultrasound/photoacoustic multi-mode imaging instrument (VEVO LAZR X, Fujifilm VisualSonics, U.S.). The hair on the hind legs of the rat was shaved. Then the rat was anesthetized by using the isoflurane inhalation (2%) at 4 L/min fresh gas flow. The anesthetized rat was placed on the platform, using a similar posture as the Raman measurements (**Figure S10a**). A dense layer of the ultrasonic coupling agent was coated on the hind limb skin surface. The ultrasonic transducer probe was placed above the limb. To avoid the possible pressure-induced position shift of the lymph node, the transducer probe did not touch the skin surface directly (~1 mm gap in between, with a layer of ultrasonic coupling agents). The vertical cross-section ultrasonic view was recorded, as shown in **Figure S10b**, which displayed the distance of SLN to the skin surface.

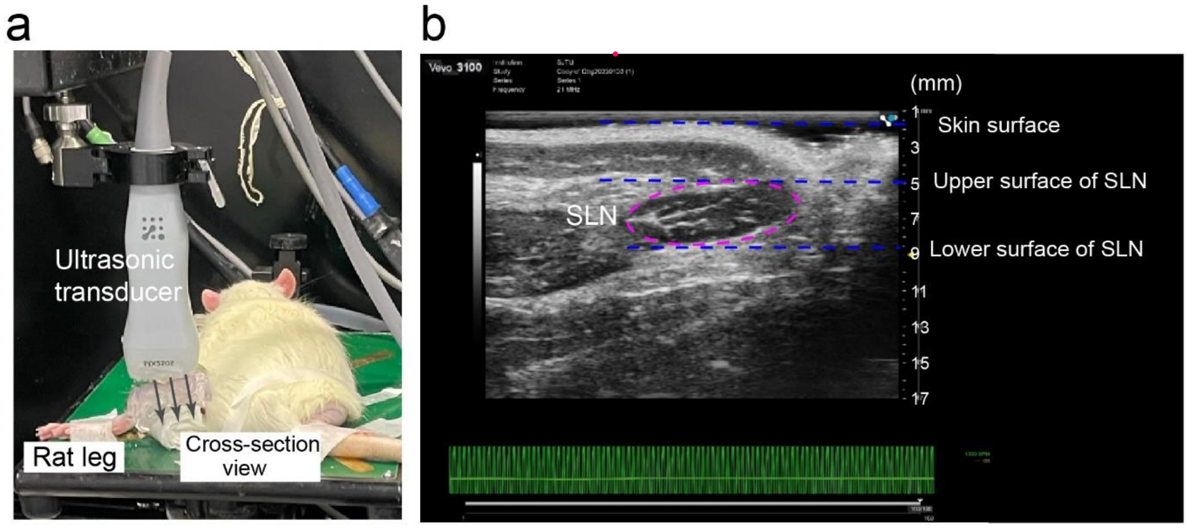

Figure S10. The sentinel lymph node (SLN) detection using the ultrasonic imaging. (a) The anesthetized rat was placed on the platform. (b) The vertical cross-section ultrasonic view showing the position and depth of SLN.

Table S4. Laser parameters for *in vivo* TRS and backscattering Raman measurements with the excitation wavelength of 785 nm. The corresponding MPE threshold was also listed according to the standards (ANSI Z 136.3, American National Standard for Safe Use of Lasers in Health Care).

	Laser power (mW)	Laser spot size	Integration time (ms)	Power density (W/cm^2)	Irradiance energy (J/cm^2)	MPE threshold (J/cm^2)
TRS	420	1.6 cm	500	0.21	0.105	1.37
Backscattering	10	100 μm	10	131	1.31	0.5

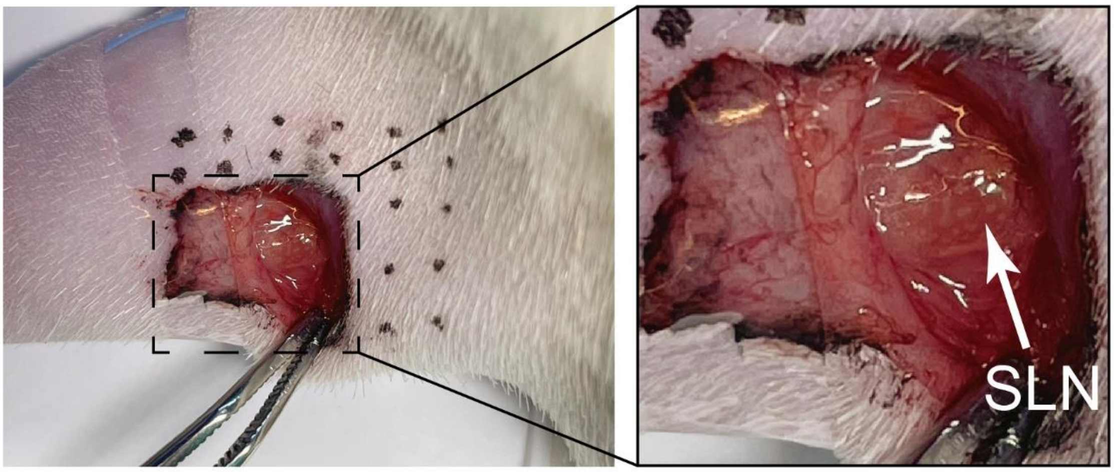

Figure S11. The exposed SLN after the removal of covered muscle tissue.

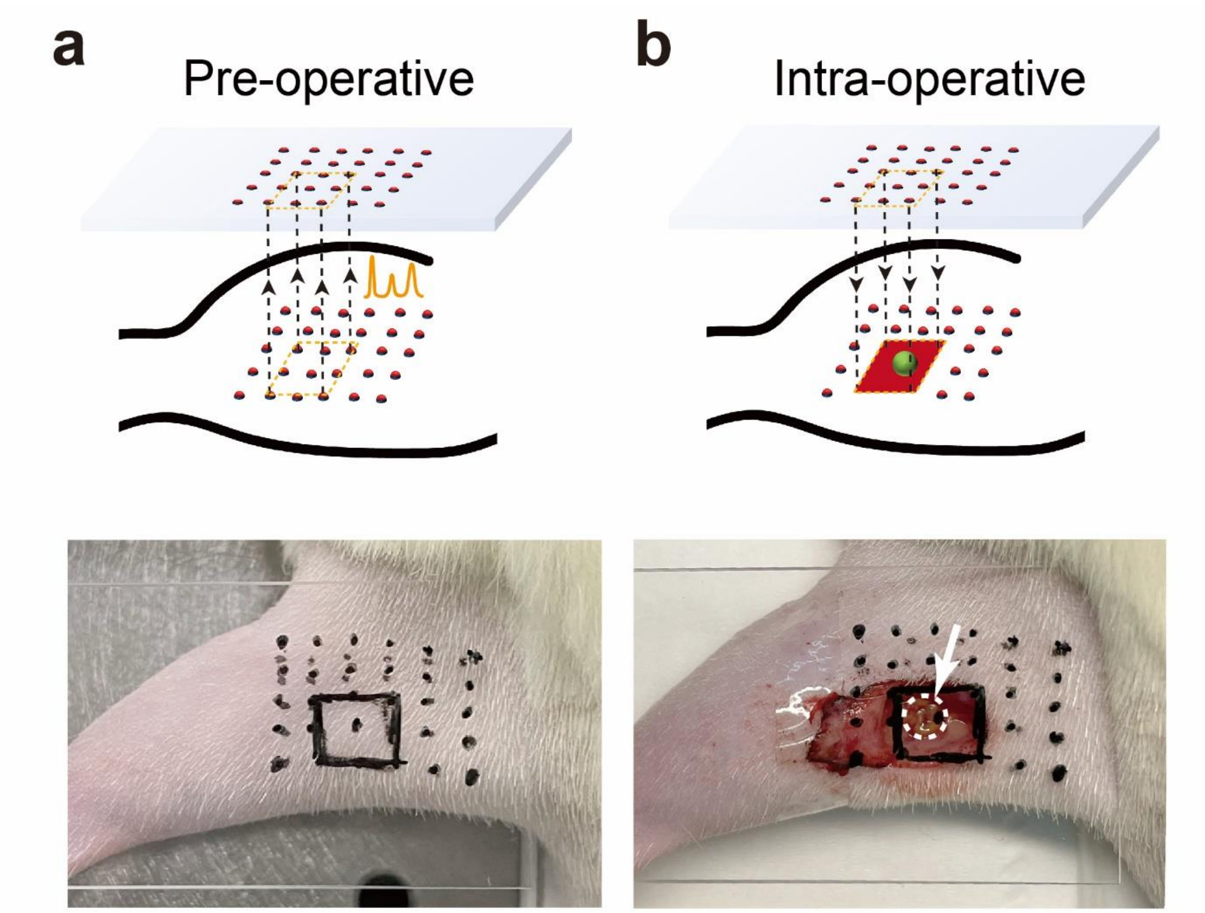

Figure S12. The accurate preoperative positioning of SLN. (a) The marks on the skin are drawn with the same size on the glass, which were used to confirm the SLN position after dissecting the skin and muscle. (b) Placed the glass slide over the skin and match the dots on the glass with the dots on the skin one by one after the skin and muscle were dissected. The frame with the highest Raman intensities on the slide completely coincides with the position of *in vivo* SLN.

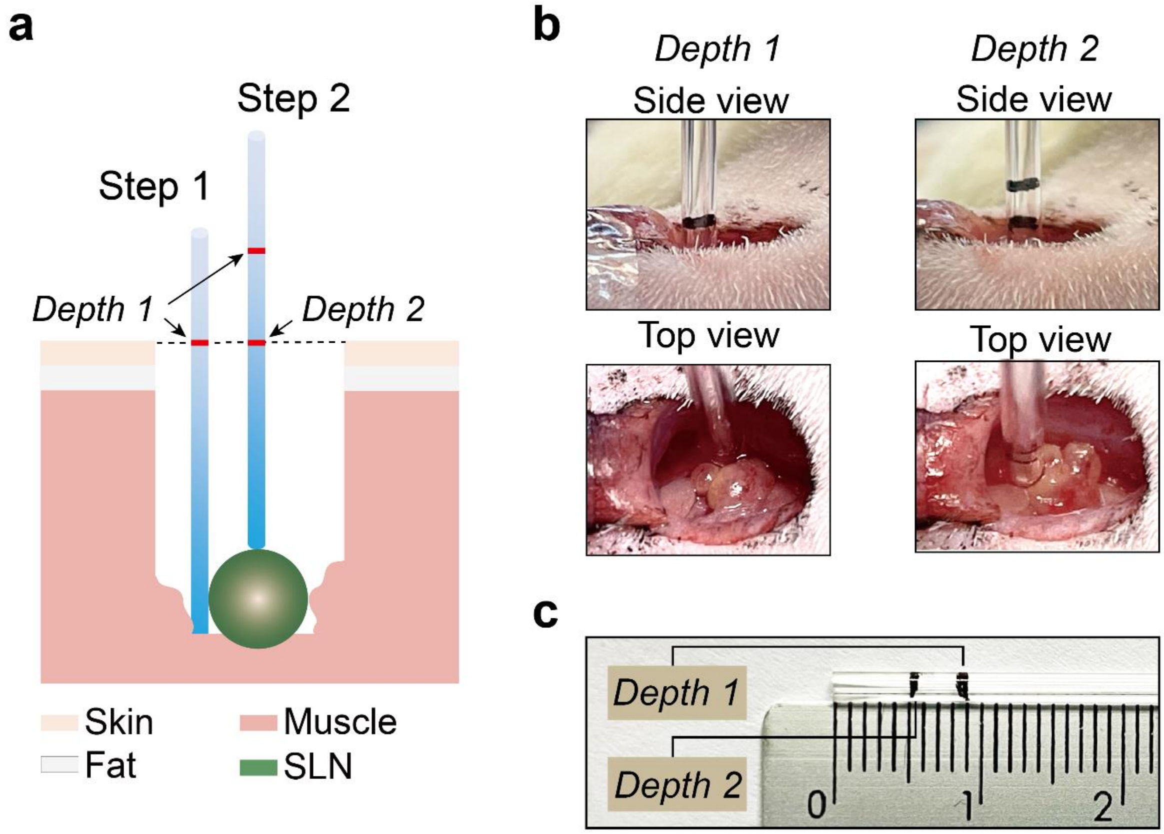

Figure S13. Depth measurements of the SLN. (a) The scheme for depth measurements. Two different depths would be obtained considering the volume of the lymph node. *Depth 1* was measured as the distance from the bottom of the lymph node to the surface. *Depth 2* was measured as the distance from the top of the lymph node to the surface. (b) The demonstration pictures of depth measurements on the side and top view. (c) The actual values of two depths. *Depth 1* was about 8.5 mm and *depth 2* was about 5.1 mm.

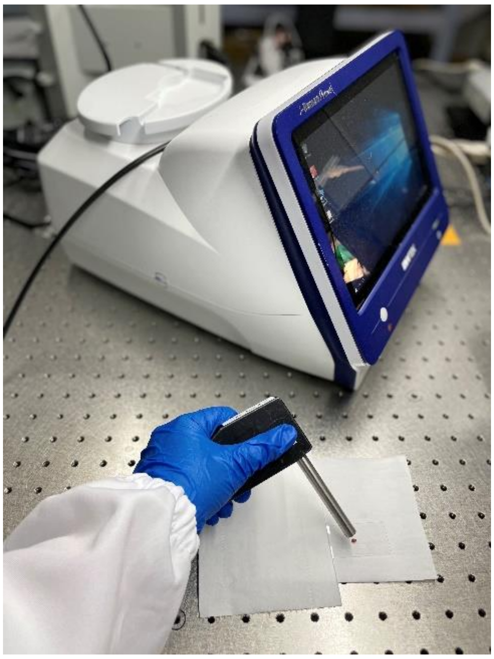

Figure S14. The backscattering Raman measurement of the resected SLN.

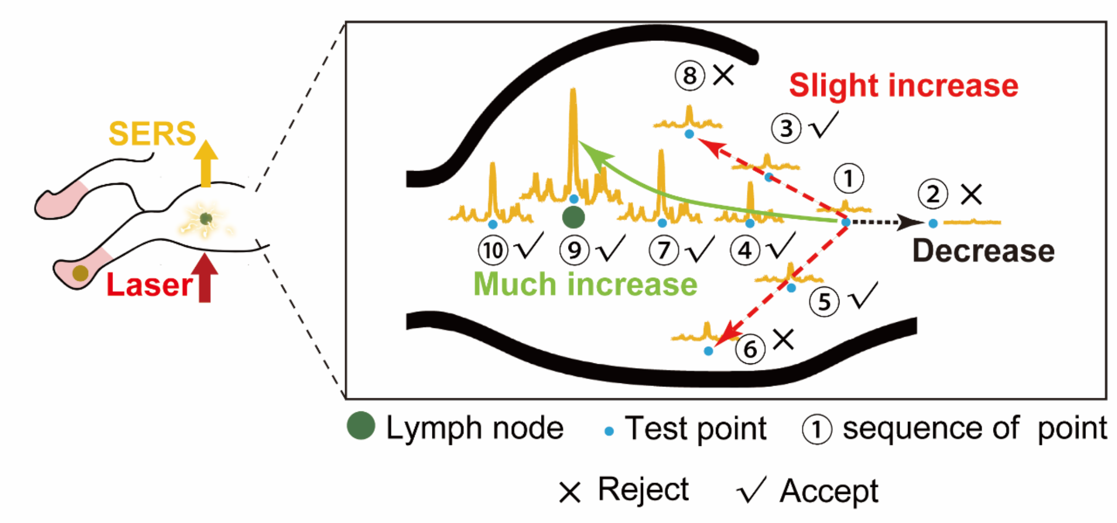

Figure S15. Scheme of non-invasive preoperative searching for the lymph node projection point. The surgeon first define a start point (① in figure) near the lymph node based on their experiences. Several directions may be explored (as point ②, ③, ④, and ⑤ shown in figure). A decrease of Raman intensity is exhibited in point ②, indicating that this direction should be rejected. An increase of intensity is observed in point ③, ④, and ⑤, meaning that these directions should be correct. Then point ⑥, ⑦ and ⑧ are tested and an obvious increase is observed in point ⑦, compared with a slight increase in point ⑥ and ⑧. So a line scanning will be conducted in the direction formed of point ①, ④ and ⑦. The projection point of lymph node could be determined according to the highest Raman intensity along this line.

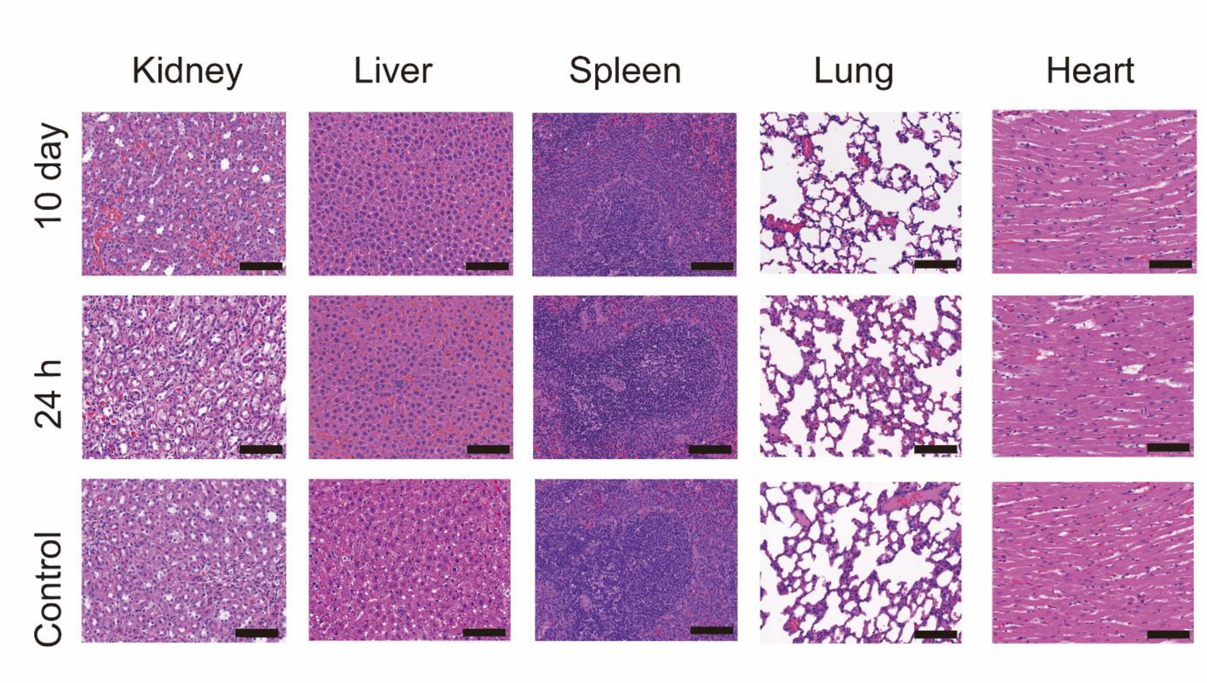

Figure S16. Histological assessment of kidney, liver, spleen, lung, and heart of the rats at 24 hours and 10 days after injection or without NP injection (control group). No significant pathological changes are found in treated rats. The SERS NP colloids, consisting of 100 μL of 0.5 nM NPs dispersed in a 1% PVP saline solution were injected into the foot pads of the rat's left and right hind legs. The scale bars are 100 μm.
